# Structure of a fully assembled γδ T cell antigen receptor

**DOI:** 10.1038/s41586-024-07920-0

**Published:** 2024-08-15

**Authors:** Benjamin S. Gully, João Ferreira Fernandes, Sachith D. Gunasinghe, Mai T. Vuong, Yuan Lui, Michael T. Rice, Liam Rashleigh, Chan-sien Lay, Dene R. Littler, Sumana Sharma, Ana Mafalda Santos, Hariprasad Venugopal, Jamie Rossjohn, Simon J. Davis

**Affiliations:** 1 Infection and Immunity Program and Department of Biochemistry and Molecular Biology, Biomedicine Discovery Institute, Monash University, Clayton, Victoria Australia; 2 Medical Research Council Weatherall Institute of Molecular Medicine, Radcliffe Department of Medicine, John Radcliffe Hospital, University of Oxford, Oxford, UK; 3 Medical Research Council Translational Immune Discovery Unit, John Radcliffe Hospital, University of Oxford, Oxford, UK; 4 Institute of Infection and Immunity, Cardiff University School of Medicine, Heath Park, Cardiff, UK

**Keywords:** Signal transduction, Cryoelectron microscopy

## Abstract

T cells in jawed vertebrates comprise two lineages, αβ T cells and γδ T cells, defined by the antigen receptors they express—that is, αβ and γδ T cell receptors (TCRs), respectively. The two lineages have different immunological roles, requiring that γδ TCRs recognize more structurally diverse ligands^1^. Nevertheless, the receptors use shared CD3 subunits to initiate signalling. Whereas the structural organization of αβ TCRs is understood^2,3^, the architecture of γδ TCRs is unknown. Here, we used cryogenic electron microscopy to determine the structure of a fully assembled, MR1-reactive, human Vγ8Vδ3 TCR–CD3δγε_2_ζ_2_ complex bound by anti-CD3ε antibody Fab fragments^4,5^. The arrangement of CD3 subunits in γδ and αβ TCRs is conserved and, although the transmembrane α-helices of the TCR-γδ and -αβ subunits differ markedly in sequence, packing of the eight transmembrane-helix bundles is similar. However, in contrast to the apparently rigid αβ TCR^2,3,6^, the γδ TCR exhibits considerable conformational heterogeneity owing to the ligand-binding TCR-γδ subunits being tethered to the CD3 subunits by their transmembrane regions only. Reducing this conformational heterogeneity by transfer of the Vγ8Vδ3 TCR variable domains to an αβ TCR enhanced receptor signalling, suggesting that γδ TCR organization reflects a compromise between efficient signalling and the ability to engage structurally diverse ligands. Our findings reveal the marked structural plasticity of the TCR on evolutionary timescales, and recast it as a highly versatile receptor capable of initiating signalling as either a rigid or flexible structure.

## Abstract (web summary)

Cryogenic electron microscopy determines the structure of a fully assembled, MR1-reactive, human Vγ8Vδ3 TCR–CD3δγε_2_ζ_2_ complex bound by anti-CD3ε antibody Fab fragments.

## Main

αβ and γδ T cells each span around 500 million years of vertebrate evolution, underscoring their important and non-overlapping immune functions^7^. αβ T cells survey the intracellular milieu of target cells via recognition of specific peptide fragments complexed with classical major histocompatibility complex (MHC) molecules, as well as lipids and metabolites presented, respectively, by non-classical MHC proteins, including CD1 and MR1 (ref. ^7^). Conversely, γδ T cells, which comprise a distinct T cell lineage with an important role in tumour and mucosal immunity, recognize a variety of structurally diverse ligands. For example, γδ T cell receptor (TCR) ligands include CD1, MR1, stress-induced MHC I-like molecules and non-MHC-like ligands, including butyrophilin and butyrophilin-like molecules^1^.

Despite these differences in ligand specificity, both TCRs consist of equivalent subunits: a ligand-binding module—that is, the TCR-αβ or -γδ heterodimer—and non-covalently associated signal-transducing dimers (CD3-ζ_2_, -δε and -γε). However, the γδ TCR exhibits structural differences in the constant (C) C-γ and C-δ domains versus the equivalent regions of the αβ TCR, and the position of an interchain disulfide bond differs, suggesting that γδ TCRs might form signalling complexes different to αβ TCRs^8,9^. The cryo-electron microscopy (cryo-EM) structures of fully assembled unligated and peptide/MHC (pMHC)-bound αβ TCRs were recently determined^2,3,6^, revealing the principles of αβ TCR assembly and showing that αβ TCRs are apparently rigid structures. We explored whether all TCRs share these properties or vary in organization, by determining the structure of a γδ TCR using cryo-EM.

## Expression and purification of a γδ TCR

Given the large repertoire of γδ TCRs, and the diversity of their ligands, an important consideration was which γδ TCR to investigate. Although Vγ9Vδ2 TCR-expressing T cells are the largest γδ T cell subset in human peripheral blood, much remains unknown about how these receptors engage their ligands to induce activation. We chose to focus on a Vγ8Vδ3 γδ TCR (called G83.C4) restricted to the highly conserved MHC-I-related molecule, MR1 (refs. ^4,10^). Given that the CD3 components assemble alongside the constant regions of the γδ TCR, it can be expected that the structural features of the Vγ8Vδ3 γδ TCR will generally reflect those of other γδ TCR assemblies incorporating different combinations of Vγ and Vδ domains. However, the well-characterized biophysical and structural properties of αβ and γδ TCRs interacting with MR1 afforded the opportunity to compare the reactivities of αβ and γδ TCRs interacting with a shared ligand^4,11^. Importantly, whereas αβ TCR–MR1 complexes adopt a ‘classical’ end-to-end docking mode in the manner of all αβ TCR–pMHC interactions^7^, some γδ TCRs deviate from this paradigm—for example, binding underneath or to the side of the antigen-binding platform of their MHC-I-like ligands^4,10,12^. The MR1-reactive G83.C4 γδ TCR and the AF-7 αβ TCR conform to this general pattern, binding to the side and to the top of the antigen-presenting cleft of MR1, respectively^4^.

**Extended Data Fig. 1:**
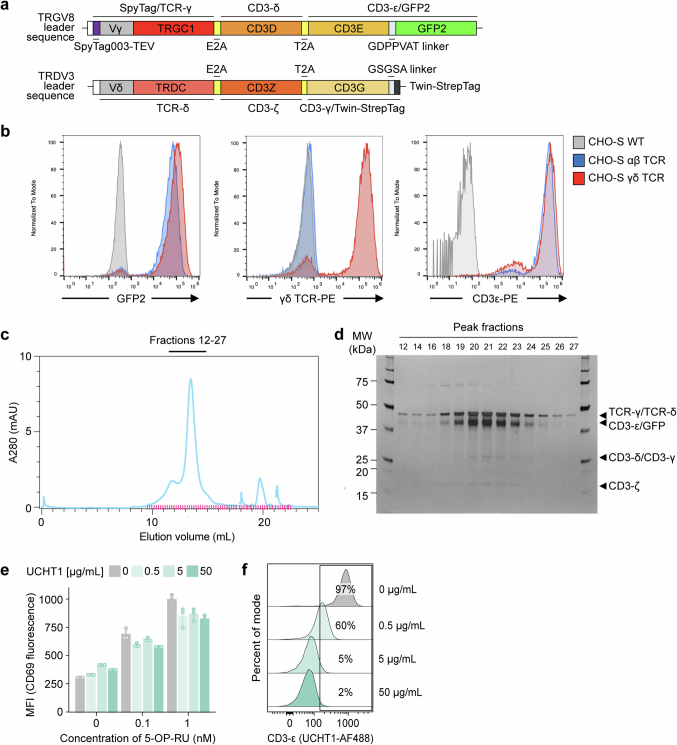
Purification of the fully assembled γδ TCR and effects of UCHT1 Fab binding on signaling. **a**, Schematic representation of the constructs used for G83.C4 γδ TCR expression. **b**, Flow cytometric validation of TCR expression in CHO-S γδ TCR-expressing cells (red) versus CHO-S WT cells (grey), or CHO-S cells used for GPa3b17 αβ TCR expression^3^ (blue). Expression was verified via GFP2 expression (left panel), anti-γδ TCR antibody staining (middle panel) and anti-CD3ε antibody staining (right panel). **c**, Analytical size-exclusion chromatography of purified G83.C4 γδ TCR. Fractions collected are labelled in pink. **d**, SDS-PAGE analysis of fractions from (c), boiled under reducing conditions. This is a single gel from one experiment. **e**, Mean fluorescence intensity (MFI) of CD69 expression by G83.C4 TCR-transduced Jurkat T-cells co-cultured with C1R cells expressing WT levels of MR1, treated with different amounts of 5-OP-RU in the presence of the indicated concentrations of UCHT1 Fab. Circles represent n = 3 treated cultures from a single experiment representative of two independent experiments. Error bars represent +/− SD. **f**, FACS plots showing fluorescent UCHT1 antibody staining of cells pre-incubated with increasing concentrations of UCHT1 Fab, confirming saturating Fab binding at the higher concentrations assayed. The experiment shown is based on the staining of a single culture (n = 1) and is representative of two independent experiments.

To express the MR1-reactive human G83.C4 γδ TCR^4^, two polycistronic constructs comprising full-length complementary DNAs encoding all six subunits of the receptor—separated by viral 2A ribosome-skipping sites and with the CD3-ε chain tagged with GFP2 to monitor receptor expression and purification—were cloned separately into lentiviral vectors (Extended Data Fig. 1a). The γδ TCR was purified from detergent-solubilized membranes prepared directly from lysed Chinese hamster ovary (CHO) cells transduced with both lentiviruses as described previously^3^, using a Twin-StrepTag attached to the CD3-γ subunit to avoid the purification of complexes lacking this subunit^13,14^. The receptor, expressed at the surface of CHO cells, bound strongly to anti-γδ TCR and anti-CD3ε antibodies, showing that it was correctly folded (Extended Data Fig. 1b). The G83.C4 γδ TCR yielded monodisperse, stable, purified complexes as confirmed by size-exclusion chromatography (Extended Data Fig. 1c), obviating the need for cross-linking. The presence of all six γδ TCR subunits was confirmed using SDS–polyacrylamide gel electrophoresis (SDS–PAGE), with typical yields in the order of around 1.9 mg l^−1^ cultured cells, testifying to the quality and stability of the purified G83.C4 γδ TCR (Extended Data Fig. 1d).

## Structural overview

**Fig. 1:**
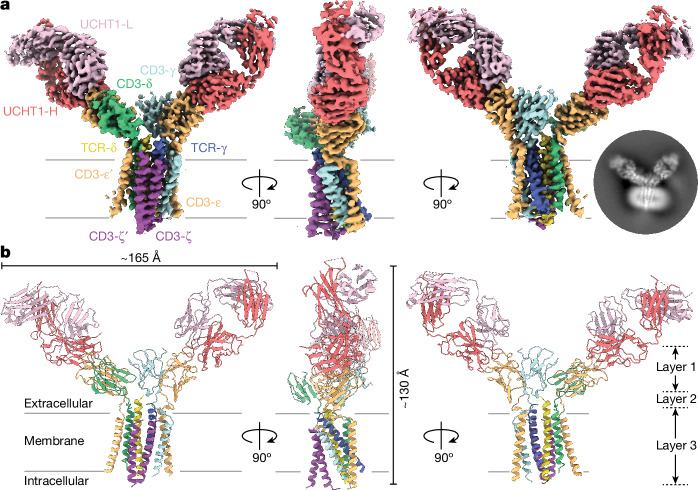
Overall structure of the fully assembled γδ TCR. **a**, Overview of the 3.01 Å consensus cryo-EM map of the G83.C4 γδ TCR bound by UCHT1 antibody Fab fragments and viewed parallel to the plane of the membrane, with the inset showing a representative reference-free, two-dimensional class average from an equivalent orientation for reference. **b**, Ribbon representation of the subunits of the G83.C4 γδ TCR, individually colour coded: TCR-δ (yellow), TCR-γ (blue), CD3-ε (orange), CD3-δ (green), CD3-γ (cyan), CD3-ζ (purple) and UCHT1 Fab heavy and light chains (red and pink, respectively); membrane boundaries are indicated by black lines. Approximate complex dimensions are 165 × 130 Å^2^. The three layers refer to distinct regions of protein contact forming the assembly.

**Extended Data Fig. 2:**
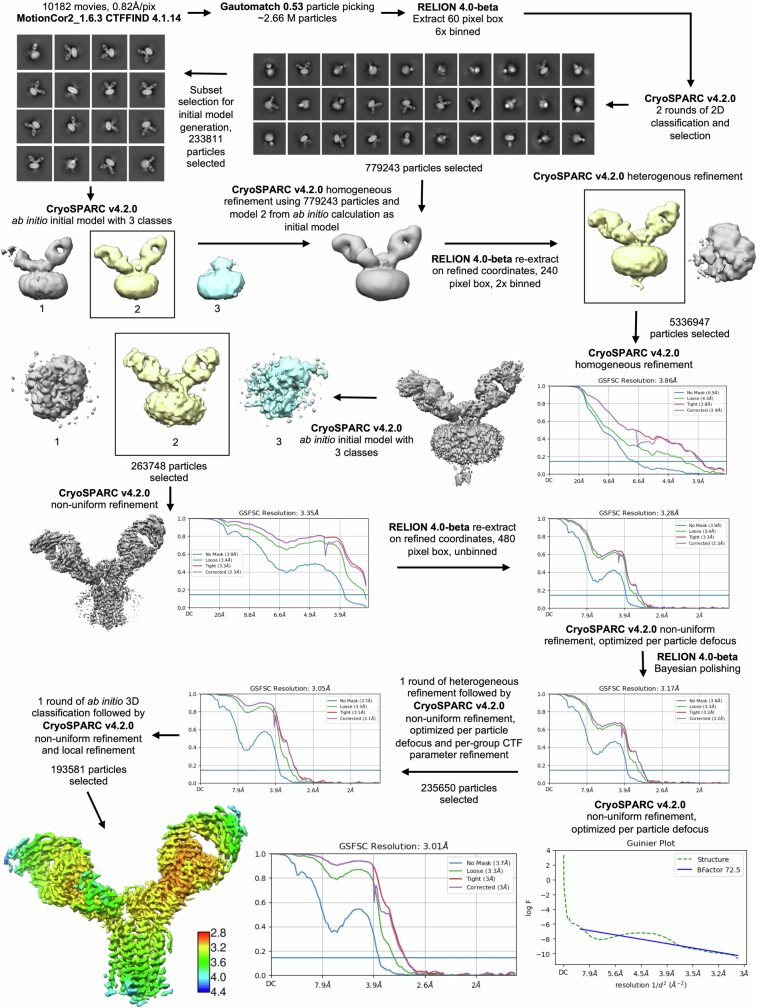
Cryo-EM analysis of the G83.C4 γδ TCR–UCHT1 Fab complex. Selected 2D class averages and data processing workflow for the G83.C4 γδ TCR–UCHT1 complex reconstructions and iterative processing optimization, are shown. Gold-standard FSC curves calculated from two independently refined half-maps indicate an overall resolution of 3.01 Å at FSC = 0.143. The local resolution-filtered display of resolution (Å) coloured from highest resolution (red) to lowest resolution (blue) is also shown.

**Extended Data Fig. 3:**
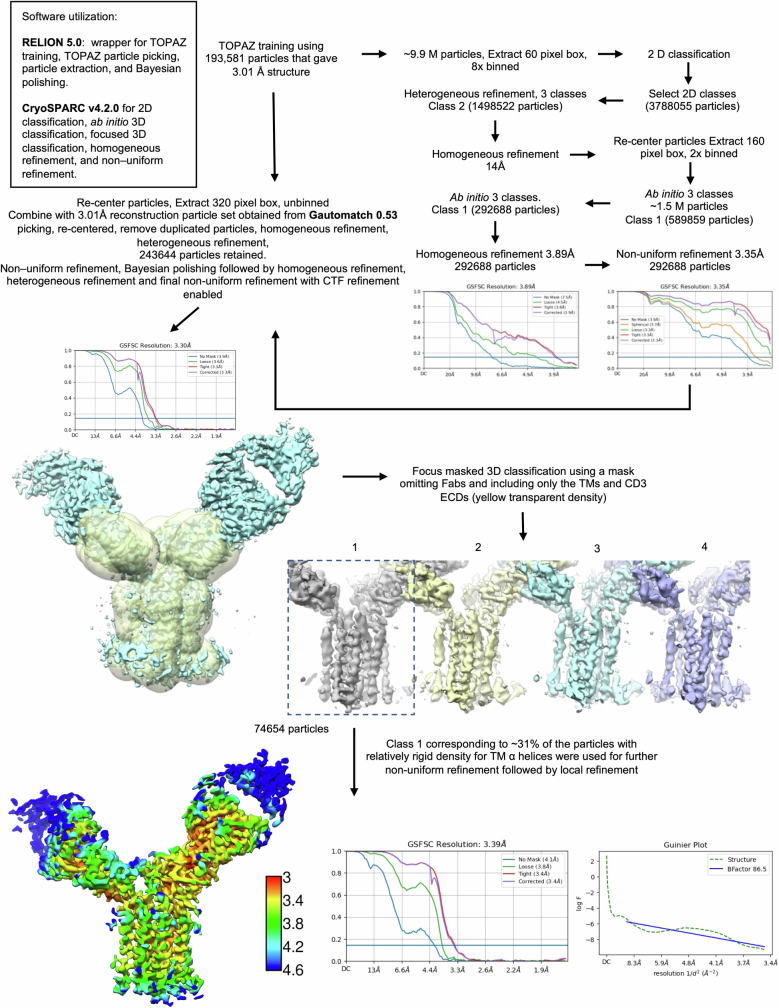
Cryo-EM processing workflow for the TM-focused G83.C4 γδ TCR reconstruction. Data processing workflow for the TM-focused G83.C4 γδ TCR–UCHT1 complex reconstruction and iterative processing optimization, are shown. Gold-standard FSC curves calculated from two independently refined half-maps indicate an overall resolution of 3.39 Å at FSC = 0.143. The local resolution-filtered display of resolution (Å) coloured from highest resolution (red) to lowest resolution (blue) is also shown.

**Extended Data Fig. 4:**
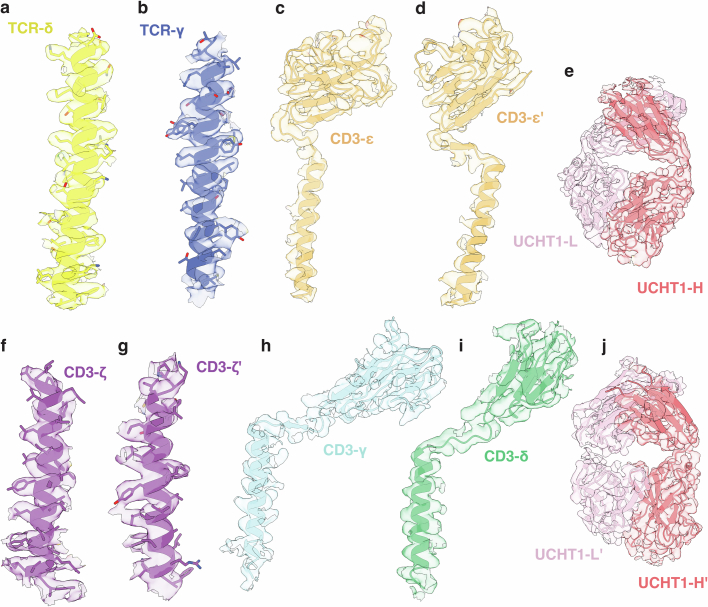
Density-to-model agreement of the G83.C4 γδ TCR–UCHT1 Fab complex in the consensus map. **a**-**j**, Density corresponding to the TCR-δ, TCR-γ, CD3-ε, CD3-δ, CD3-ε′, CD3-γ, CD3-ζ, CD3-ζ′, UCHT1 and UCHT1′ polypeptides are shown for the global 3.01 Å reconstruction. Chains are coloured as in Fig. 1; map threshold is ~0.13.

**Extended Data Fig. 5:**
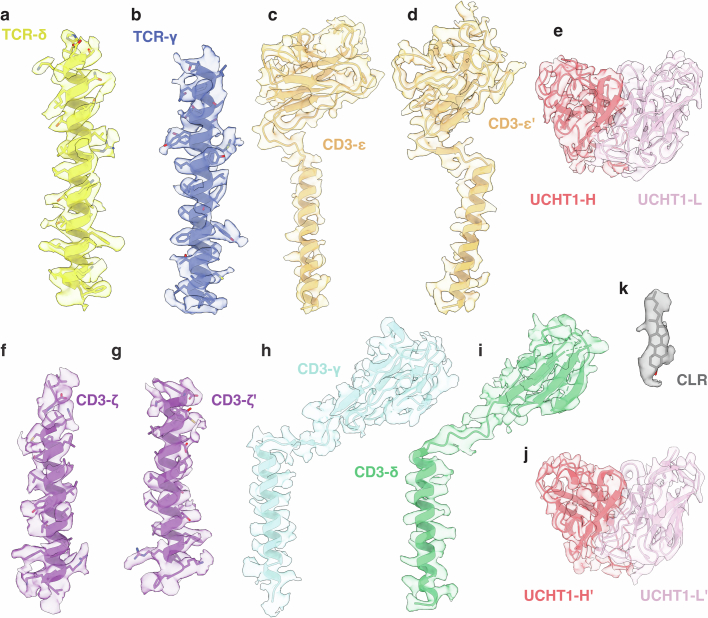
Density-to-model agreement of the G83.C4 γδ TCR–UCHT1 Fab complex in the TM-focused map. **a**-**j** Density corresponding to the TCR-δ, TCR-γ, CD3-ε, CD3-δ, CD3-ε′, CD3-γ, CD3-ζ, CD3-ζ′, UCHT1 and UCHT1′ polypeptides and, **k**, for the transmembrane-located cholesterol-like molecule (CLR), are shown for the TM-focused 3.39 Å reconstruction. Chains are coloured as in Fig. 1; map threshold is ~0.13.

Purified detergent-solubilized G83.C4 γδ TCRs were attached to Fab fragments of the well-studied CD3ε-specific antibody UCHT1 (ref. ^5^), to aid high-resolution structural analysis. Because anti-CD3 Fab fragments^15,16^ and UCHT1 antibodies^17^ are reported to affect T cell activity, we used signalling assays to confirm that the Fab fragments we prepared were inert (Extended Data Fig. 1e,f). The purified complex was vitrified and imaged by single-particle cryo-EM, yielding a 3.01 Å consensus map (Extended Data Fig. 2 and Extended Data Table 1). Overall, the G83.C4 γδ TCR–UCHT1 complex comprised a bilobed, V-shaped structure formed by two UCHT1 Fab fragments that each engaged a CD3 extracellular domain (ECD), converging above the transmembrane (TM) helical assembly (Fig. 1). Additional refinement centred on the TM core yielded a focused map with a global resolution of 3.39 Å and well-resolved TM and membrane-proximal regions, improving on the overall map in these regions (Extended Data Fig. 3 and Extended Data Table 1). High-resolution features across the two maps enabled many side-chains to be assigned for the two models, with ambiguous regions left unmodelled (Extended Data Figs. 4 and 5). The G83.C4 γδ TCR–UCHT1 complex consensus reconstruction confirmed that TCR-γδ heterodimers associated 1:1:1:1 with CD3-δε, -γε and -ζζ dimers (Fig. 1a), indicating that our method of tagging the CD3-γ chain had avoided differential CD3 stoichiometries^13,14^. Unexpectedly, the TCR-γδ ECDs were poorly resolved in the consensus map despite clear signal, indicating that this region is notably mobile, in marked contrast to detergent-solubilized αβ TCRs^2,3^ but analogous to Fab regions of B cell receptor (BCR) complexes^18^. The arrangement of the CD3-δε and -γε heterodimer ECDs was highly reminiscent of the αβ TCR which, in that receptor, places the TCR-αβ heterodimer in a tilted position^2,3,6^ but, in the γδ TCR, probably facilitates only receptor assembly. Each CD3-ε subunit was bound by a UCHT1 Fab fragment, creating a pseudosymmetric, V-shaped structure roughly 165 Å in width and 130 Å in length (Fig. 1b). The UCHT1 Fab fragments were readily interpretable within the consensus map (Fig. 1a), in contrast to the pMHC-αβ TCR–UCHT1 Fab complex^3^. Attempts to determine the structure of the γδ TCR in the absence of UCHT1 Fab fragments were unsuccessful. Comparison of Fab-bound and -unbound αβ TCRs has previously shown that UCHT1 Fab binding is not accompanied by conformational rearrangements in the CD3 or other subunits of the αβ TCR^2,3^. The conserved CD3 arrangement extended to the TM regions where, despite large TCR-γδ and -αβ TM region sequence differences, equivalent interactions formed. Limited density was observed for the cytosolic regions of the TCR-γδ and CD3 subunits, as in the case of the apo form of the αβ TCR^2^, indicating that these regions are highly mobile. In the following analysis of the structure, we work from the membrane outwards—that is, from layer 3 out to layers 1 and 2 (ref. ^3^) (Fig. 1b)—emphasizing, in particular, differences and similarities in the organization of γδ and αβ TCRs^2^. We then consider the implications of ligand-binding domain mobility for γδ T cell responsiveness, having investigated its effects on receptor signalling using chimeric constructs.

## γδ TCR TM assembly

**Fig. 2:**
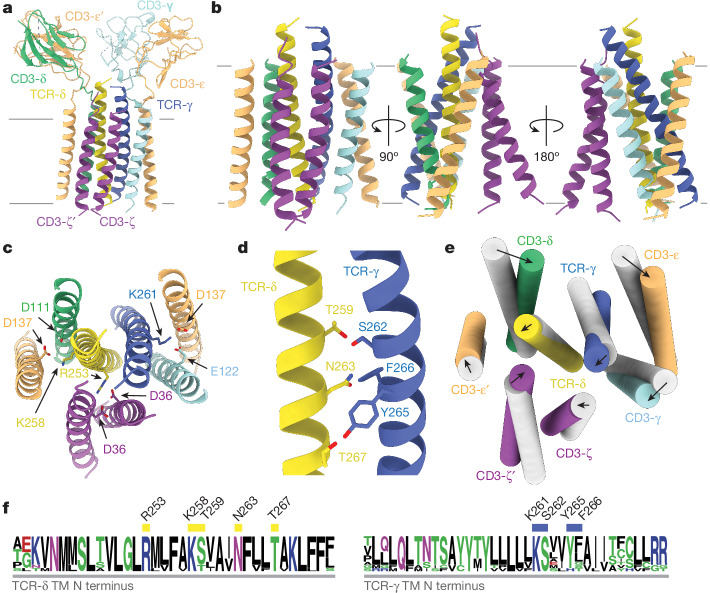
Interactions in the TM helical bundle of the γδ TCR. **a**, Overview of the 3.39 Å TM-focused model of the G83.C4 γδ TCR. **b**, Ribbon representation of the G83.C4 γδ TCR TM region and the TCR-γδ, CD3-δε, CD3-γε and CD3-ζζ helical dimers comprising layer 3 of the receptor assembly. **c**, Conserved charged TM contacts (the view is the same as in **b** (left), rotated by 90° along an axis in the plane of the page). **d**, TM contacts of the TCR-γ and -δ subunits. **e**, Comparison of the organization of the TM helices in the apo αβ TCR (PDB 6JXR, grey) versus their counterparts in the γδ TCR. Arrows and arrowheads represent changes in position between the two complexes, measuring below 5 Å throughout. **f**, Sequence logos for the γδ TCR TM regions showing the conservation of key TM contacts, highlighted by the yellow (TCR-δ) and blue (TCR-γ) rectangles. Subunits are coloured as in Fig. 1, with dashed lines indicating unmodelled regions of the structure. Residues are numbered throughout according to the full-length (that is, unprocessed) sequence.

**Extended Data Fig. 6:**
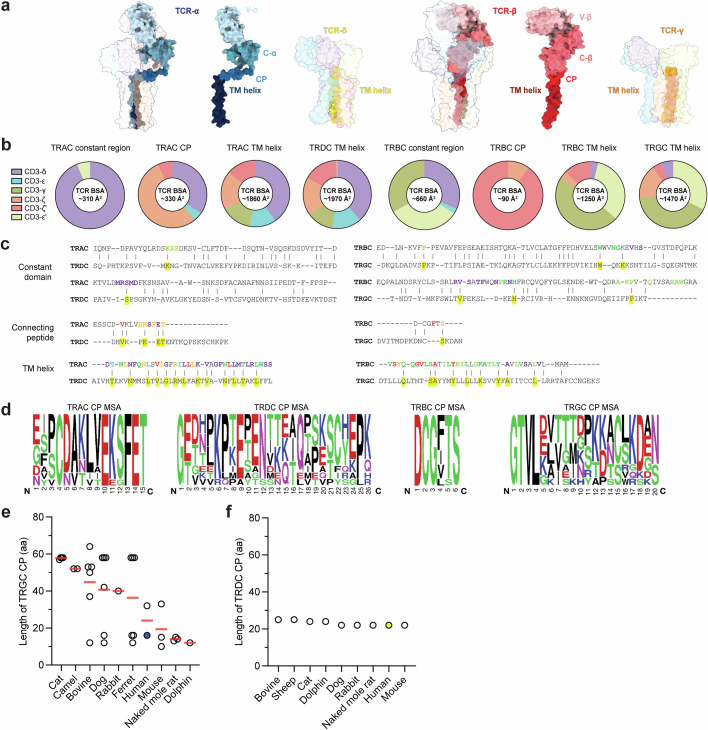
Bound surface areas (BSAs) for αβ and G83.C4 γδ TCRs. **a**, Structural depiction of TCR-α and -β chains in the αβ TCR complex^3^ and TCR-γ and TCR-δ chains in the TM-focused γδ TCR structure, with interacting regions labelled. **b**, BSA (%) in each of the αβ TCR regions, and the TM helices of the γδ TCR only (due to flexibility of the TCR-γδ ECDs and CPs), coloured with respect to the fraction of contact made with the indicated CD3 subunits. **c**, Alignments of α and β, and γ and δ constant regions, and CP and TM helical region sequences. The TCR-α and -β subunit/CD3 contacts shown in bold are coloured according to the individual CD3 subunit contact made. Conserved TCR-δ and -γ subunit contacts are highlighted in yellow. **d**, Poor evolutionary conservation of CP sequences of TCR-α, -β, -δ, and -γ subunits. Sequence logos illustrate the degree of amino acid conservation in exon 2 of mammalian sequences [TCR-α, -δ sequences: 11 species; TCR-β, -γ sequences: 10 species (C1 sequence only used)]. The structure of the pMHC-bound GPa3b17 TCR (PDB 7PHR) was used for the comparisons in (a,b). **e**, Variation in TCR-γ CP sequences across mammalian species encoded by *TRGC* genes. Highlighted in dark blue is the human *TRGC1*-encoded CP of the γδ TCR studied here. Mean length is represented by red bars. **f**, Length and variation of CP sequences encoded by mammalian *TRDC* genes. Highlighted in yellow is the human *TRDC*-encoded CP.

The G83.C4 γδ TCR was anchored to the membrane via an eight-chain helical TM core (Fig. 2a) that was well resolved in the TM-focused map (Extended Data Fig. 3) and comprised a single-pass TM helix from each chain of the TCR-γδ, CD3-δε, CD3-γε and CD3-ζζ dimers, in the manner of the αβ TCR^2,3,6^. The sequences of TCR-γ and -δ TM regions exhibit limited similarity to their αβ counterparts—that is, 36 and 32% for the δ/α and γ/β TM regions, respectively (Extended Data Fig. 6). Despite this, the eight-chain helical TM core is assembled around the centrally located TCR-γ and -δ chains, wholly in the manner of the αβ TCR (Fig. 2b). Whereas, for the αβ TCR, the TM regions of the CD3 dimers pack around the centrally located TCR-αβ TM helical core partly due to interlocking interactions in the linker region^2,3,6^, in the γδ TCR, similar interactions with the TCR-γδ subunits are absent and the assembly relies heavily on interactions involving strictly conserved, charged TM region residues^19^ (Fig. 2c). Lys258 and Arg253 of TCR-δ form salt bridges with Asp111 of CD3-δ and Asp137 of CD3-ε′ in the CD3-δε heterodimer, and with the two Asp36 residues of the CD3-ζζ dimer, respectively, replicating the interactions of the TCR-α chain. Similarly, Lys261 of the TCR-γ chain forms salt bridges with Glu122 of CD3-γ and Asp137 of CD3-ε in the CD3-γε heterodimer, matching those formed by the TCR-β chain. The degree of interdigitation of TM helices varies across the membrane in the manner of the αβ TCR, with ‘splayed’ CD3 heterodimer TM region interactions in the outer, but not inner, leaflet, favouring neutralization of the TM region charged residues (Fig. 2b,c). Similarly, the CD3-ζ chains are also asymmetrically arranged, with the CD3-ζ′ chain displaced from the rest of the TM region assembly, especially towards the cytosol (Fig. 2b,c).

The positions of the TCR-γ and -δ TM helices relative to one another are fixed by a pair of interactions close to the centre of the membrane (Fig. 2d), between Ser262 and Thr259 and between Tyr265 and Thr267 of TCR-γ and -δ, respectively. The TCR-αβ heterodimer is similarly stabilized by membrane-buried hydrogen bonds, specifically Tyr272 and Thr245 of TCR-β and -α, respectively^3^. Sandwiched between these bonds, Asn263 of TCR-δ interacts with Phe266 and Tyr265 of TCR-γ, which slightly repositions the TCR-γ and -δ TM helices. Coupled with small movements of the CD3 ECDs, this creates coordinated rigid body movements in the TM regions resulting in minor shifts (roughly 2 Å) in the positioning of each of the TM helices of the eight-helix TM core of the γδ and αβ TCRs (Fig. 2e). The residues involved in TCR-γ and -δ TM region interactions are highly conserved across species, like the charged residues that direct interaction of these subunits with the CD3 heterodimers (Fig. 2f). The hydrophobic residues that surround the charge clusters are also invariant or conservatively replaced (Fig. 2f). Accordingly, the general mode of γδ TCR TM region packing observed here is likely to be conserved across species.

**Extended Data Fig. 7:**
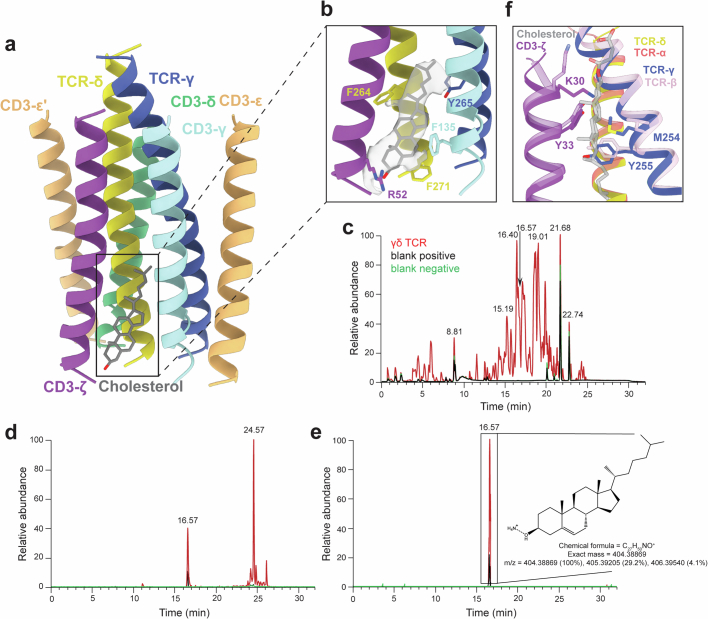
Cholesterol modeling and mass spectrometric analysis of the G83.C4 γδ TCR. **a**,**b** Ribbon representations of the G83.C4 TCR-γδ-CD3 TM region showing the possible location of a cholesterol-like moiety proximal to the inner membrane leaflet and C-termini of the CD3-ζ, CD3-γ, and TCR-γ TM helices, capped by R52 of CD3-ζ, with map threshold ~0.13 in (b). **c**, Mass spectrometry-derived chromatographic feature consistent with the ammonium adduct of cholesterol (positive mode *m/z* = 404.38667 – 404.39071) at 16.6 min (arrow) in a purified G83.C4 γδ TCR sample and a positive control cholesterol sample, but not the negative control sample (**d**). **e**, In-source fragmentation consistent with the loss of ammonia and water was also observed (fragment ion *m/z* = 369.34973 – 369.35343) at 16.6 min in the G83.C4 γδ TCR sample and positive control, but not the negative control sample. **f**, Region corresponding to the outer leaflet cholesterol binding site of the αβ TCR (PDB 8ES7), which appears to be blocked by Met254 of the TCR-γ TM helix in the γδ TCR.

Alongside the TCR-δ TM region on the inner leaflet side of the membrane, both the consensus and TM core-focused maps exhibited density suggestive of a coordinated cholesterol molecule, located within a pocket formed by Phe264 of TCR-δ, Tyr265 of TCR-γ and Phe135 of CD3-γ, that is capped on the cytosolic side by Arg52 of CD3-ζ (Extended Data Fig. 7a,b). Mass spectrometric analysis of the purified complex identified chromatographic features and adducts consistent with the presence of cholesterol (Extended Data Fig. 7c–e). A second potential cholesterol-binding site towards the outer leaflet was empty, even though it is lined by hydrophobic residues like those in the equivalent, sterol-occupied region of the αβ TCR^3,6^ (Extended Data Fig. 7f). Within this site, however, Lys30 of CD3-ζ is repositioned towards Met254 of TCR-γ, probably preventing the outer leaflet site from binding cholesterol in the γδ TCR. Because the potential cholesterol-binding site of the γδ TCR corresponds to the second site in the αβ TCR on the inner leaflet side, we tentatively modelled it into the cryo-EM map^20^ (Extended Data Fig. 7a,b). Although a Vγ9Vδ2 TCR was reported not to bind cholesterol^21^, this seems unlikely to be the case for the Vγ8Vδ3 TCR investigated here, suggesting that cholesterol could have important structural and/or regulatory roles^21,22^. Despite the notable divergence of the TM sequences of the TCR-γδ and TCR-αβ subunits, the architecture of this region is highly conserved and reliant on key charged residues shared across TCR subtypes and species^23^.

## CD3 subunit ECD interactions

**Fig. 3:**
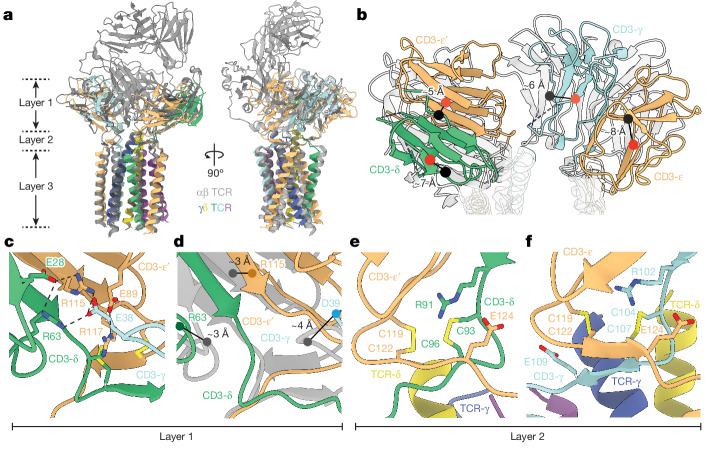
Interactions involving CD3 ECDs in the G83.C4 γδ TCR. **a**, Comparison of the organization of the CD3 heterodimers in the TM-focused model of the G83.C4 γδ TCR and an αβ TCR (PDB 7PHR). The TCR-γ and TCR-δ subunits of the γδ TCR, and the CD3 subunits, are coloured as in Fig. 1, with the αβ TCR shown in greyscale. **b**, Comparison of CD3 ECD displacement within the two complexes, with the centres of mass of the CD3 heterodimers coloured red for the γδ TCR and black for the αβ TCR, showing shifts of up to around 8 Å in positions of the subunits between the complexes. Both complexes were solved bound to UCHT1 Fab fragments, allowing comparison. **c**,**d**, Layer 1 interactions between CD3-δε and -γε heterodimers in the αβ (**c**) and γδ (**d**) TCRs. In **d**, the view of the αβ TCR presented in **c** is shown in greyscale, and because the main-chain position in the region of Glu38 in the γδ TCR could not be confidently modelled, movement in the region of the adjacent residue, Asp39, is shown. **e**,**f**, Stabilizing effects of interactions in the regions of the layer 2 vicinal cysteines of CD3-δε (**e**) and CD3-γε (**f**).

The tilted geometry of the CD3-γε and -δε ECDs of the αβ TCR, imposed by two structural layers comprising the CD3 ECDs (layer 1, Fig. 1b) and their connecting peptides (CPs; layer 2, Fig. 1b), which allows close association of their TM regions^3^, is reprised in the γδ TCR, as expected (Fig. 3a). However, the TCR-γδ subunits do not contribute contacts to either layer in the γδ TCR, in contrast to the TCR-αβ subunits of the αβ TCR. The structures of the folded ECDs of the CD3-εδ and -εγ heterodimers are conserved between the γδ and αβ TCRs, each stabilized by well-characterized β-strand G aromatic ladders^24,25^. However, although the structural underpinnings of the layer 1 interactions are similar, they are not identical: the centres of mass of the CD3-δε and -γε ECDs of the γδ TCR differ by about 5–8 Å from those of the αβ TCR (Fig. 3b). In the γδ TCR, the CD3-δε and -γε heterodimer ECDs have scarcely any identifiable pairwise interactions. For the αβ TCR, the heterodimers are held in position via multiple contacts, including a three-way interaction involving an AB loop glutamate in CD3-γ (Glu38) and arginines in CD3-δ (Arg63) and CD3-ε (Arg115; Fig. 3c) supported by a salt-bridge and H-bond involving Glu28 of CD3-δ and Tyr113 of CD3-ε, respectively, and interactions of the CD3-εγ and -εδ heterodimer ECDs with the base of the TCR-αβ constant regions. These interactions position CD3-εγ next to TCR-β and CD3-εδ alongside TCR-α, producing the tilted arrangement of TCR-αβ relative to the membrane^2,3^. The side-chains creating this three-way interaction between the CD3-γ AB loop, CD3-δ and CD3-ε ECDs are poorly resolved in the γδ TCR, but main-chain displacements in this region (Fig. 3d) suggest that this interaction might not occur, perhaps due to the absence of stabilizing contacts with the TCR-γδ constant domains.

**Extended Data Fig. 8:**
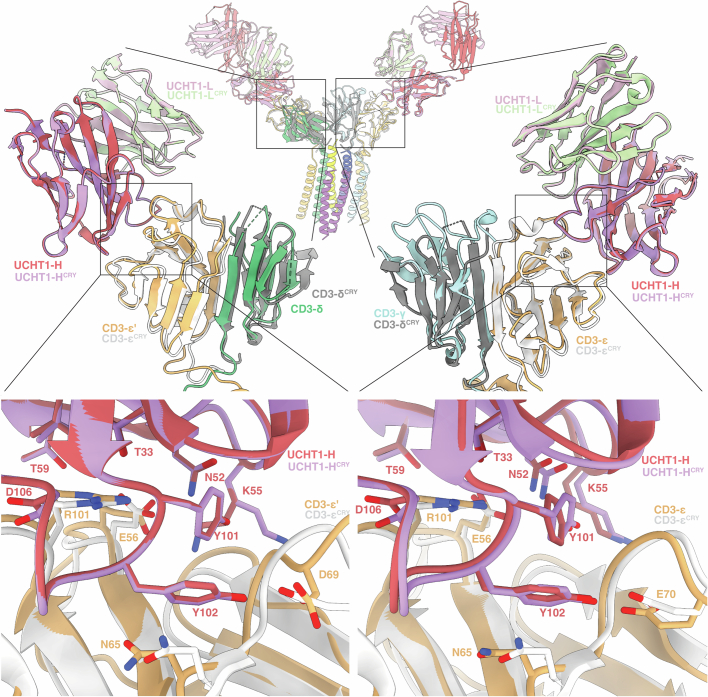
Comparison of the UCHT1 interfaces. Structural alignment of the CD3-δε and CD3-γε extracellular domains of the G83.C4 γδ TCR complexed with UCHT1 Fab fragments, versus the soluble CD3-δε-UCHT1 complex structure (CRY; PDB 1XIW) reveals minimal change at the respective interfaces.

The positioning of the CD3-δε and -γε ECDs in the γδ TCR relies also on a complex network of layer 2 contacts within their membrane-proximal CPs. This includes coordinated intra- and interchain contacts involving the highly conserved vicinal CXXC cysteines of the CD3-δ, -ε and -γ chains^26^. The CD3-δε heterodimer ECD is stabilized by disulfide bonds formed by Cys93 and Cys96 of CD3-δ, and by Cys119 and Cys122 of CD3-ε (Fig. 3e,f). The second cysteine within each CXXC motif resides within short β-strands, capped by arginine at their N termini—for example, Arg91 in CD3-δ and Arg102 of CD3-γ—and glutamate at the C termini—for example, Glu124 of CD3-ε (Fig. 3e,f). These β-strands, which ensure that the CD3 ECDs are packed against the top of the TM helix bundle, are highly conserved in αβ TCRs^2,3,6^ and explain the residual positional stability of the CD3 heterodimers in the absence of layer 1 and 2 contributions from the TCR-γ and -δ subunits. The two UCHT1 Fab fragments bound the FG loops of both CD3-ε chains of the γδ TCR, as observed in the complex of a single-chain UCHT1 fragment bound to CD3-δε (ref. ^24^) (Extended Data Fig. 8). Accordingly, the arrangement of the CD3 heterodimers was broadly unchanged relative to the αβ TCR.

## γδ TCR extracellular domain mobility

**Fig. 4:**
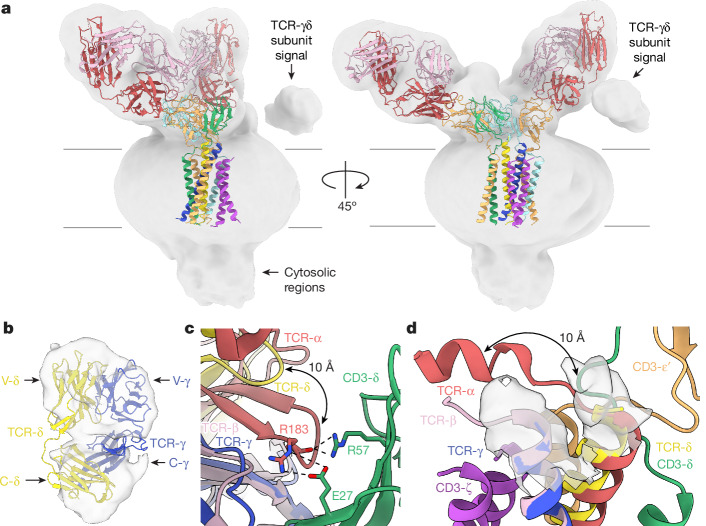
Structural heterogeneity of the G83.C4 γδ TCR. **a**, A Gaussian-filtered version of the 3.01 Å consensus G83.C4 γδ TCR reconstruction (grey volume), showing the clear signal for the TCR-γδ heterodimer ECD, which was variably positioned relative to the CD3 assembly, shown in ribbon format and coloured as in Fig. 1. **b**, Additional data processing allowed the reconstruction of a low-resolution map of the G83.C4 TCR-γδ heterodimer ECD (grey surface), at the cost of knowing the position of the remainder of the receptor. Positioning of the previously determined structure of a soluble, chimeric G83.C4 TCR-γδ heterodimer ECD (comprising C-α and C-β domains; PDB 7LLI) within the reconstruction was undertaken using Chimera. **c**, Superposition of a known TCR-γδ heterodimer ECD^13^ with the TCR-αβ heterodimer ECD (PDB 7PHR) within the CD3 complex indicates that a shortened TCR-δ constant domain DE loop, relative to that of TCR-α, would remove key contacts to CD3-δ. **d**, The longer CPs of the G83.C4 γδ TCR are clearly mobile, although the reconstruction shows a 10 Å shift in the positions of the TCR-α and -δ CPs when aligned with the αβ TCR. γδ TCR subunits are coloured as in Fig. 1a; TCR-α and -β subunits are coloured red and pink, respectively.

**Extended Data Fig. 9:**
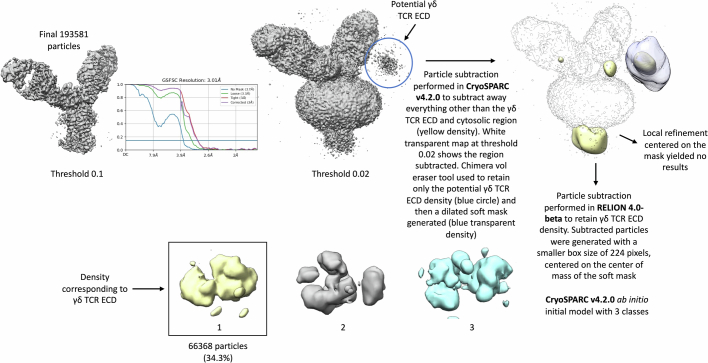
Additional analysis of the G83.C4 TCR-γδ ECD arrangement in the γδ TCR. Data processing workflow for reconstruction of the G83.C4 TCR-γδ ECD. Following optimized processing of the initial reconstruction, the flexible region of the G83.C4 γδ TCR required particle subtraction and *ab initio* model generation to enable a modest-resolution reconstruction of this part of the sample to be obtained.

An unexpected finding in the initial reconstruction and consensus map was the mobility of the TCR-γδ subunit ECDs (Fig. 4a and Extended Data Fig. 9). The strong signal for the ECDs enabled signal subtraction and local refinement to yield an ab initio reconstruction of the TCR-γδ subunit ECD heterodimer (Fig. 4b). Although the small size of the heterodimer hindered further processing, the reference-free convergence of the TCR-γδ ECD reconstruction confirmed the conformational mobility of this region relative to the CD3 assembly. This contrasts with the equivalent region of the αβ TCR, which, as judged by multiple cryo-EM analyses, is apparently rigidly positioned^2,3,6^. In this sense, the TCR-γδ ECD heterodimer is instead analogous to the Fab regions of BCR complexes^18^.

**Extended Data Fig. 10:**
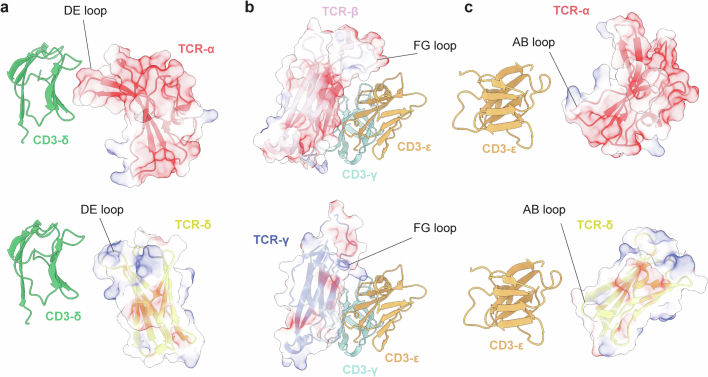
Structural comparisons of the TCR-αβ and -γδ subunit constant domains in the context of the CD3 subunits. Comparison of γδ TCR constant region loops (PDB 4LFH) in the setting of the CD3δγε_2_ζ_2_ complex following alignment with an αβ TCR constant chain domain (PDB 7PHR). TCR electrostatics are shown as a surface and individual chains are coloured as in Fig. 1. **a**, DE loop; **b**, FG loop; and **c**, AB loop. The shorter inter-strand loops of the γδ TCR constant region would likely reduce CD3 contact, favouring TCR-γδ ECD mobility.

The contrasting mobilities of the TCR-γδ and -αβ heterodimers result from key C region differences. First, the αβ and γδ C domains share little sequence identity (roughly 12 and 25% for the C-δ/C-α and C-γ/C-β comparisons, respectively) and are structurally divergent. Notably, automated comparisons (https://search.foldseek.com/search) rank, on average, as the domains most like C-γ and C-δ structures, 24- and >100-antibody CH or CL domains above C-β and C-α domains, respectively (although the results obtained for C-δ varied for other algorithms). C-α and C-γ domains also have altered surface electrostatics and lack the prominent FG loop of the C-β domain reported to be important in αβ T cell function (for example, ref. ^27^), alongside other loop structure differences^28^. Superposition of the TCR-γδ ECD heterodimer with TCR-αβ in the αβ TCR complex indicated that steric clashes do not prevent γδ TCR assembly in the manner of the αβ TCR (Extended Data Fig. 10). Instead, it shows that the structural differences have the effect of removing key CD3-δε- and -γε-interacting residues. Specifically, the DE loop of C-δ is three residues shorter than the corresponding C-α loop, preventing C-α-like contacts with the CD3-δ ECD—that is, of Arg183 of C-α with Glu27 and Arg57 of CD3-δ (Fig. 4c). Similarly, a charge reversal in C-γ prevents contact of Trp260 with Tyr36 of CD3-γ and Leu90 of CD3-ε in the manner of C-β. Second, the membrane-proximal C-γ and C-δ CPs are 14 and 13 residues longer, respectively, in the γδ TCR compared with the equivalent CPs of the αβ TCR^27,29^. Although the density was weak in this region, limiting modelling of the γδ TCR CPs, the consensus and local maps suggested that the CPs extend directly away from the membrane, in contrast to the TCR-α and -β CPs (Fig. 4d). We also note that the human *TRGC2* gene encodes an extra 16 amino acids in the TCR-γ CP and removes the membrane-proximal interchain disulfide^27,29^. Moreover, other mammals express several C-γ CP isoforms, with most mammalian C-γ genes encoding CP segments of equivalent length or longer than that of human *TRGC1* (ref. ^30^) (Extended Data Fig. 6e). However, the overall size of γδ TCR complexes might in most instances be constrained by the relatively short, largely invariant-length CPs encoded by the single *TRDC* genes (Extended Data Fig. 6f). Finally, the base of the TCR-δ C domain is glycosylated^10,31^ and, very probably, also the C-γ CP regions^30^, working against the formation of a compact αβ TCR-like assembly.

## γδ and αβ TCR reactivities towards a shared ligand

**Fig. 5:**
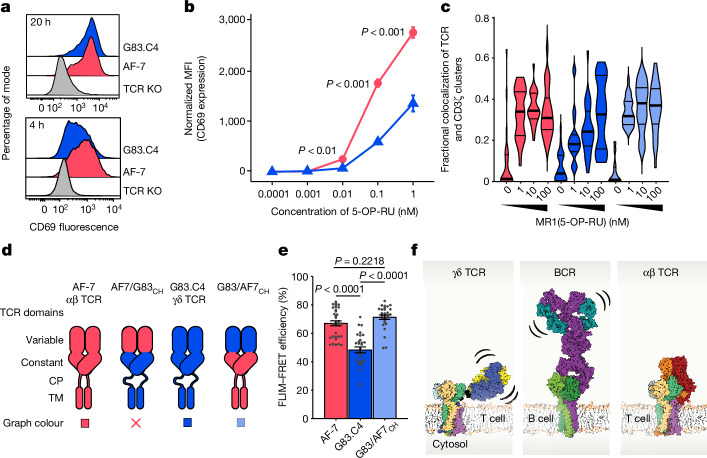
Effects of γδ and αβ TCR flexibility on TCR ligand sensitivity. **a**, Representative flow cytometry plots depicting the level of CD69 expression by either TCR knockout Jurkat T cells or AF-7 WT or G83.C4 WT TCR-transduced Jurkat T cells, cocultured for either 20 h (top) or 4 h (bottom), with C1R cells treated with 1 nM 5-OP-RU. **b**, Mean fluorescence intensity (MFI) of CD69 expression by AF-7 and G83.C4 TCR-transduced Jurkat T cells cocultured for 4 h with C1R cells treated with 5-OP-RU at a range of concentrations (0.0001–1.0 nM). **c**, TCR phosphorylation analysis based on single-molecule imaging of TCR and phospho-CD3ζ clusters and the degree of their colocalization (Methods), following stimulation of Jurkat T cells expressing the AF-7 TCR (red), G83.C4 TCR (dark blue) or a chimeric TCR (G83/AF7_CH_, light blue; see **d**), on ICAM1 or ICAM1 + MR1(5-OP-RU)-bearing bilayers. **d**, Cartoon schematic of the G83.C4 and AF-7 WT and chimeric TCR constructs (AF7/G83_CH_ and G83/AF7_CH_). **e**, FLIM–FRET efficiency for Jurkat T cell transductants labelled with fluorescent donor MR1(5-OP-RU)-Atto 594 and fluorescent acceptor UCHT1 Fab-Alexa Fluor 647. **f**, Schematic depicting the structures of the antigen receptors, including depictions of the γδ TCR (this work), the IgM BCR^18^ and the αβ TCR^2^. **b**,**e**, *P* values were calculated using Student’s *t*-test (**b**) or one-way analysis of varance with Tukey’s multiple-comparisons test (**e**). Error bars represent ±s.d. (**b**) or s.e.m. (**e**). **c**, Thick horizontal lines indicate the median, and thin lines the quartiles. **a**–**c**,**e**, Data are representative of *n* = 2 cocultures in either two independent experiments (**a**), two independent experiments analysing *n* = 3 cocultures each (**b**), two independent experiments each including *n* ≥ 12 cells (**c**) or two independent experiments each including *n* ≥ 14 cells (**e**).

**Extended Data Fig. 11:**
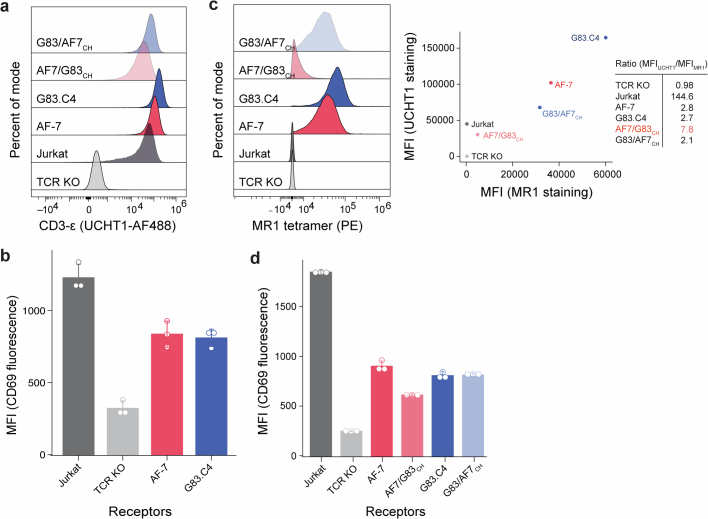
Characterization of cell lines used for functional assays. **a**, FACS plots showing CD3 expression levels (as measured by UCHT1-AF488 staining) on parental Jurkat T-cells (dark grey), TCR-knockout (KO) Jurkat T-cells (light grey), or TCR-KO Jurkat T-cells transduced with AF-7 (dark red), G83.C4 (dark blue), AF7/G83_CH_ (light red), and G83/AF7_CH_ (light blue) TCRs. Data shown are from a single culture (n = 1) from one experiment. **b**, MFI quantification of CD69 expression levels on transductants activated for 4 h on plate-bound OKT3, as determined by flow cytometric analysis for WT (dark grey), TCR-KO (light grey), and AF-7-expressing (dark red) and G83.C4-expressing (dark blue) Jurkat T-cells. Circles represent measurements from n = 3 co-cultures from one experiment, representative of two independent experiments. Bar charts and error bars represent mean +/− SD. **c**, FACS plots of MR1-PE tetramer staining of Jurkat transductants (left panel), as in (a), and quantification of MR1 tetramer MFI plotted against UCHT1 MFI from (a) (right panel). Quantification of the ratio of UCHT1 and MR1 tetramer MFI is depicted in the table inset. Data are from a single culture (n = 1) from one experiment. **d**, MFI quantification of CD69 expression levels on transductants activated for 4 h on plate-bound OKT3, as determined by flow cytometric analysis of WT (dark grey) and TCR-KO (light grey) Jurkat T-cells, and Jurkat T-cells expressing AF-7 (dark red), G83.C4 (dark blue), AF7/G83_CH_ (light red), and G83/AF7_CH_ (light blue) TCRs. Circles represent measurements from n = 3 co-cultures from one experiment. Bar charts and error bars represent mean +/− SD.

Our choice of the Vγ8Vδ3 G83.C4 TCR allowed us to compare the reactivities of a γδ and an αβ TCR—that is, AF-7—with a shared ligand, MR1, presenting the bacterial metabolite 5-(2-oxopropylideneamino)-6-d-ribitylaminouracil (5-OP-RU). AF-7 is a semi-invariant TCR, expressed by mucosal-associated invariant T cells, that binds MR1(5-OP-RU) with a dissociation constant (1.75 μM) comparable to that of G83.C4 (0.57 μM)^4^. Jurkat T cells lacking endogenous TCRs and expressing, instead, the G83.C4 γδ and AF-7 αβ TCRs (Extended Data Fig. 11a) were incubated with MR1-expressing C1R cells pulsed with 5-OP-RU, with CD69 upregulation used as a marker of their activation. By a late time point (20 h post stimulation), both sets of cells had responded equally well to MR1(5-OP-RU) stimulation; at 4 h post stimulation, however, AF-7-expressing Jurkat T cells produced higher levels of CD69 compared with cells expressing the G83.C4 TCR (Fig. 5a and Supplementary Fig. 1). At the early time point, G83.C4 TCR-expressing cells required about tenfold more MR1 ligand to reach the same level of activation as AF-7 TCR-expressing cells (Fig. 5b). This difference was not due to an intrinsic signalling deficiency in G83.C4-expressing cells, because both sets of cells expressed similar levels of CD69 in response to plate-bound anti-CD3ε OKT3 antibodies (Extended Data Fig. 11b). This trend suggested that larger differences might be observed during proximal TCR signalling. Single-molecule imaging of cells interacting with ICAM1-bearing supported lipid bilayers presenting MR1(5-OP-RU) showed that 10–100-fold larger amounts of ligand were required by G83.C4 TCR-expressing cells to produce levels of receptor phosphorylation matching those of AF-7 expressors (Fig. 5c).

**Extended Data Fig. 12:**
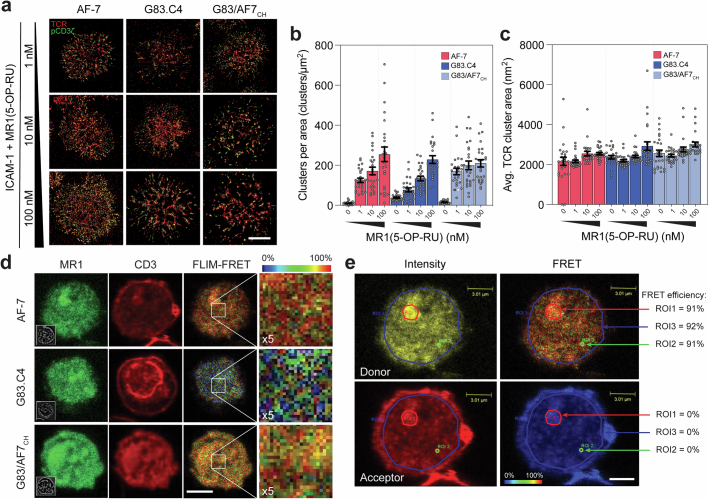
Single-molecule and FLIM-FRET imaging of cells expressing WT and chimeric TCRs. **a**, Representative dSTORM images generated for cells expressing AF-7 (left panels), G83.C4 (middle panels), and chimeric G83/AF7_CH_ receptors (right panels) when stimulated with increasing amounts of MR1-5-OP-RU on an SLB and stained with anti-CD3-AF647 (red) and anti-pCD3ζ-AF568 (green) antibody. Scale bar = 5 µm. **b**,**c** Number and average area of TCR clusters as quantified by DBSCAN. Data are from two independent experiments each using n ≥ 12 cells. Error bars represent ± SEM. **d**, Confocal images generated for fluorescent donor MR1(5-OP-RU)-Atto 594 (green, left panels)- and fluorescent acceptor anti-UCHT1 Fab-AF647 (red, center panels)-presenting Jurkat T-cells, expressing AF-7 (top panels), G83.C4 (middle panels), or chimeric G83/AF7_CH_ TCRs (bottom panels). A heatmap of FLIM-FRET efficiency between TCR and MR1 monomers (right panels), with colour representing highest to lowest efficiencies (red to blue) is also shown. Close-ups of selected regions in the confocal images are shown as x5 enlarged images. Data are representative of two independent experiments each including n ≥ 14 cells. Scale bar = 5 µm. **e**, FLIM-FRET efficiency is independent of local TCR density. FRET efficiencies across various regions of interest (ROI), including punctate regions of corresponding donor and acceptor fluorescence and the entire cell area, are shown. The ROI FRET efficiencies (%) in the FRET heatmap are also indicated. Scale bar = 3 μm.

To investigate the structural basis of these effects, we generated chimeric TCR constructs in which the variable regions of the G83.C4 and AF-7 TCRs were swapped, called G83/AF7_CH_ and AF7/G83_CH_ (Fig. 5d). The chimeric constructs reached the cell surface, albeit slightly less efficiently than the wild-type (WT) proteins (Extended Data Fig. 11a). For AF7/G83_CH_ we observed both impaired MR1(5-OP-RU) binding as a function of CD3 expression (Extended Data Fig. 11c) and reduced signalling on plate-bound OKT3 (Extended Data Fig. 11d), so this construct was not analysed further. Cells expressing G83/AF7_CH_, however, bound MR1(5-OP-RU) comparably (Extended Data Fig. 11c) and responded similarly to plate-bound OKT3 (Extended Data Fig. 11d). Notably, the G83/AF7_CH_ receptor produced levels of TCR phosphorylation substantially higher than those generated by G83.C4 and comparable to those produced by AF-7 (Fig. 5c and Extended Data Fig. 12a–c). The transfer of γδ variable regions to αβ TCRs has previously been shown also to enhance TCR signalling in response to MHC-like molecules in a murine setting^32^.

Finally, to directly link the signalling differences between WT and chimeric TCRs to their flexibility, we used Förster resonance energy transfer and fluorescence lifetime imaging microscopy (FLIM–FRET). For this, we labelled WT and chimeric TCRs with fluorescent MR1(5-OP-RU) ligand in solution, and measured FLIM–FRET efficiency (Extended Data Fig. 12d) between the ligand and TCR-bound, anti-CD3ε UCHT1 Fab fragments. Because G83.C4-, AF-7- and G83/AF7_CH_-expressing cells bound MR1(5-OP-RU) equally well (Extended Data Fig. 11c), FLIM–FRET efficiency can be used as an indirect measure of the distance between the TCR-γδ or -αβ and CD3-ε subunit ECDs in the TCR assembly. We observed reduced FLIM–FRET efficiency for cells expressing the G83.C4 TCR versus those expressing AF-7 (Fig. 5e and Extended Data Fig. 12d). FLIM–FRET efficiency was insensitive to local TCR density (Extended Data Fig. 12e), implying that it measures differences in intramolecule flexibility and/or distance. Importantly, FLIM–FRET efficiency was restored in G83/AF7_CH_-expressing cells, matching the levels obtained for the AF-7 receptor (Fig. 5e). Given the similarity in the organization of the CD3 subunits and TM regions of the γδ and αβ TCRs, we interpret these data as indicating that it is the shorter CPs and greater rigidity of the AF-7 and G83/AF7_CH_ TCRs that account for the increased reactivity of T cells expressing these TCRs versus the G83.C4 TCR. An overview of the major structural differences in the three classes of antigen receptors is shown in Fig. 5f.

## Discussion

We determined the structure of a fully assembled γδ TCR. The organization of the CD3 ECDs and overall arrangement of the TM regions of the αβ and γδ TCRs are conserved. As in the case of the αβ TCR, the cytosolic regions appear to be unstructured. However, whereas the ECDs and TM regions of the αβ TCR form an apparently rigid structural unit^2,3^, its TCR-γδ CPs and constant regions ensure that the γδ TCR is especially flexible. The ECDs of the TCR-γδ heterodimer do not adopt any single arrangement, presumably allowing the γδ receptor to engage a variety of surface-immobilized ligands in different ways, as implied by recent crystallographic studies^4,10,12^. Along with extended CP regions and C domain structural differences, glycosylation of the TCR-δ C domain^10,31^ and the CP of TCR-γ^30^ is likely to have a special role in ensuring that the TCR-γδ ECD is mobile. In certain respects, the γδ TCR is more BCR than αβ TCR like, with the CPs and TCR-γδ ECDs corresponding to the hinge and Fab regions of antibodies. Consistent with this interpretation, along with the known, larger variation in CDR3 loop lengths^33^, structural comparisons were suggestive of the existence of an especially close evolutionary relationship between the TCR-γδ ECD and Fab regions (for example, ref. ^30^). The number and diversity of γδ TCR isotypes versus αβ TCRs among vertebrates previously led to the notion that the γδ TCR is the more ancient receptor and that BCRs and γδ TCRs share a direct ancestor, with the αβ TCR emerging secondarily from the γδ lineage (the so-called MHC capture hypothesis)^30^. All γδ TCRs will probably form intrinsically flexible complexes given that their organization seems to be determined by subunit interactions involving only non-clonotypic (that is, shared) regions of the receptors. This is now exemplified by the determination by Xin et al.^34^ of the structure of a Vγ9Vδ2 TCR that also exhibited very high levels of intrinsic flexibility. Their determination also of a Vγ5Vδ1 TCR structure, which forms dimers, further underscores the structural plasticity of TCRs^34^.

A common assumption has held that γδ TCR triggering would parallel that of the αβ receptor because they share CD3 signalling subunits, and the largely conserved arrangement of the CD3 subunits observed in the new structure strengthens this view. The γδ TCR is not thought to be a mechanotransducer as has been proposed for the αβ TCR^32^, or to rely on conformational rearrangements^17^. TCRs with either rigid or mobile ligand-binding subunits could be triggered by local phosphatase exclusion^35^, however, if their ligands are needed only to trap receptors in phosphatase-depleted cell contacts to initiate signalling^36^. γδ TCRs can engage their ligands with affinities comparable to those of αβ TCRs, but some γδ TCRs are known to signal poorly and some γδ T cells are hyporesponsive^10,12,32,37^. We observed substantial differences in the sensitivity of the G83.C4 γδ and AF-7 αβ receptors, effects we linked to the profoundly different mobilities of their ligand-binding domains with respect to their signalling subunits. The link between flexibility and signalling needs to be explored further, but one explanation for why T cells expressing γδ TCRs might exhibit constrained sensitivity is that the receptor occupies more conformational states and engages ligands less efficiently, in contrast to the apparently rigid organization of the αβ TCR, which seems to be optimized for binding an essentially monomorphic ligand^3^. In effect, γδ TCR organization could reflect a compromise between efficient signalling and the ability to engage structurally diverse ligands. However, it is also possible that, if the γδ receptor was to form ‘taller’ complexes owing to its extended CPs (albeit constrained by the single, relatively short TCR-δ chain CP, as we have noted), the efficiency of phosphatase exclusion would be lower and signalling reduced if, as is suggested^38^, γδ TCR triggering does depend on local phosphatase exclusion^35,39^. In each case, γδ TCR triggering may require relatively high levels of ligand expression ensuring that, during lineage commitment, development or activation, γδ T cells do not react to self-elements of their ligands in the case of, for example, Vγ9Vδ2 T cells that respond to butyrophilins^1^. The structure reported here should be helpful for re-engineering the γδ TCR and optimizing the reactivity of γδ T cells to MHC-like and other ligands in therapeutic settings.

## Methods

### Cell culture

The adherent human HEK293T cell line (ATCC, no. CRL-3216) was cultured at 37 °C, 5% CO_2_ in DMEM (Gibco, no. 41965-039) supplemented with 10% (v/v) heat-inactivated fetal bovine serum (FBS), 1 mM sodium pyruvate, 4 mM l-glutamine, 50 U ml^−1^ penicillin, 50 μg ml^−1^ streptomycin and 100 μg ml^−1^ neomycin. The adherent Chinese hamster cell line CHO-K1 (Lonza) was cultured at 37 °C, 5% CO_2_ in CHO-K1 medium—DMEM (Gibco, 10938-025), supplemented with 10% (v/v) heat-inactivated FBS, 1 mM sodium pyruvate, 4 mM l-glutamine, 50 U ml^−1^ penicillin, 50 μg ml^−1^ streptomycin and 100 μg ml^−1^ neomycin. The Chinese hamster CHO-S cell line (Gibco, no. R80007) was cultured in CHO-S medium, comprising FreeStyle CHO Expression Medium (Thermo Fisher, no. 12651-022) supplemented with 8 mM l-glutamine. Cells were maintained in suspension culture in Erlenmeyer flasks and cultured at 37 °C with rotation at 125 rpm in an atmosphere of 8% CO_2_ with 80–85% humidity.

### Lentivirus production

Stable protein expression was achieved using lentiviral transduction. Constructs were introduced into the pHR-SIN plasmid for expression under the SFFV promoter^3^. For lentivirus production, HEK293T cells were cotransfected using GeneJuice (Merck, no. 70967-6) with a single pHR-SIN plasmid and the packaging plasmids pMD.G and p8.91, as previously described^3^. The culture supernatant was collected 48–72 h post transfection, filtered using 0.45 μm PES filters (Sartorius) and used immediately to transduce mammalian cell lines, as described below.

### UCHT1 Fab production

A construct encoding the Fab fragment of the anti-human CD3ε murine antibody UCHT1 (ref. ^40^) was designed. Both Fab chains were expressed under a modified cPTPRσ signal peptide (MGILPSPGMPALLSLVSLLSVLLMGCVAGT, with the final two residues modified to introduce a KpnI restriction site), followed by the chain sequences. The Fab heavy chain was designed using the VH domain of UCHT1 (NCBI Protein, no. PH0887), followed by the C-γ1 domain of murine IgG1 (UniProt, no. P01868-1, residues 1–103), a GGS linker and a C-terminal C-Tag affinity tag (EPEA). The Fab light chain was designed using the Vκ domain of UCHT1 (NCBI Protein, no. PH0888, residues 1–107), followed by the murine constant kappa domain (UniProt, no. P01837). Constructs were introduced into the pHR-SIN plasmid and lentiviral particles for both chains were produced as described above.

For UCHT1 Fab production, 10^6^ CHO-K1 cells were transduced with 2 ml of viral supernatant for each chain and expanded. Culture medium was replaced with harvest medium (CHO-K1 medium with 1% (v/v) FBS) for 5 days. Culture supernatant was 0.22 μm filtered and applied directly to a CaptureSelect C-tagXL Affinity Matrix (Thermo Fisher, no. 943072050) pre-equilibrated in PBS pH 7.4. The affinity resin was washed in 20 column volumes of PBS pH 7.4, and the bound protein was eluted by competition in PBS pH 7.4 supplemented with 3 mM SEPEA peptide (custom-made, GenScript). Eluted protein was concentrated using a 10 kDa MWCO filter (Amicon) and purified into PBS pH 8.0 using a HiLoad 16/600 Superdex 200 pg chromatography column in an AKTAPure system (Cytiva). Fab fragment purity was further verified using SDS–PAGE.

### γδ TCR protein expression

For expression of a human γδ TCR, the previously described MR1-restricted Vγ8^+^Vδ3^+^ clone G83.C4 was used^4^. The TCR-γ chain was designed using the Vγ domain paired with the *TRGC1*-encoded C-γ domain (UniProt, no. P0CF51). The TCR-δ chain was designed using the G83.C4 Vδ domain paired with the *TRDC*-encoded Cδ domain (UniProt, no. B7Z8K6).

Two polycystronic constructs were designed, containing all chains required for the assembly of a complete TCR with TCRγδCD3γδζ_2_ε_2_ stoichiometry (Extended Data Fig. 2a and Supplementary Table 1). This was achieved making use of the viral self-cleaving peptides E2A ((GSG)QCTNYALLKLAGDVESNPGP) and T2A ((GSG)EGRGSLLTCGDVEENPGP). The full-length G83.C4 TCR-γ chain, with an N-terminal SpyTag003 sequence and TEV protease cleavage site, was encoded after the endogenous *TRGV8*-encoded leader sequence in combination with the full-length human CD3-δ (UniProt, no. P04234-1) and GFP2-fused^3^, full-length CD3-ε (UniProt, no. P07766). In a separate construct, the full-length G83.C4 TCR-δ chain was expressed with the endogenous *TRDV3*-encoded leader sequence in combination with the full-length CD3-ζ chain (UniProt, no. P20963-1) and the full-length CD3-γ chain (UniProt, no. P09693). For complex purification, a flexible linker (GSGSA) and a Twin-StrepTag (WSHPQFEK-(GGGS)_2_-GGSA-WSHPQFEK) sequence were introduced into the C terminus of CD3-γ. This design ensured purification of CD3-γ-containing complexes, because some reports have suggested that the complex can assemble in the absence of CD3-γ (ref. ^13^). All constructs were introduced into the pHR-SIN vector, as previously described^3^. For expression of the complex, CHO-S cells were transduced with both constructs. CHO-S cells (10^6^) were incubated with 3 ml of lentiviral supernatant for each construct and supplemented with CHO-S medium 24 h following transduction, to a final culture volume of 20 ml. Cells were cultured in suspension, and 10^6^ transduced cells were retransduced using the same protocol 7 days post transduction. G83.C4 TCR expression was validated using flow cytometry to measure GFP2 expression, as well as surface antibody staining, with either PE-conjugated antihuman CD3ε antibody (clone UCHT1, Biolegend, no. 300408, diluted 1:10) or PE-conjugated antihuman TCR-γδ antibody (clone B1, Biolegend, no. 331210, diluted 1:40; Extended Data Fig. 1b). Cells were stained with fluorescent antibodies for 60 min at 4 °C in PBS supplemented with 0.05% (w/v) NaN_3_, washed once in PBS 0.05% (w/v) NaN_3_ and fixed in PBS 0.05% (w/v) NaN_3_ supplemented with 1% (w/v) paraformaldehyde. Samples were analysed using an Attune NxT flow cytometer (Thermo Fisher).

### Purification of the γδ TCR

G83.C4 TCR-expressing CHO-S cells were grown in CHO-S medium in 2 l Erlenmeyer flasks to a final density of 3–4 × 10^6^ cells ml^−1^; 30 × 10^9^ cells were harvested from ten 1 l cultures by centrifugation at 560*g* for 15 min at 4 °C. Cells were washed twice in ice-cold PBS and cell pellets frozen at −80 °C until further use. Cell pellets were then thawed and resuspended in 30 mM Tris-HCl pH 8.0 and 750 mM NaCl, supplemented with 10 μg ml^−1^ DNAseI (Roche), a minimum of 2.5 U ml^−1^ benzonase nuclease (Sigma) and cOmplete protease inhibitor cocktail (EDTA-free, Roche). All purification steps were conducted at 4 °C unless otherwise stated. Cells were disrupted at 5,000 pounds per square inch using a 1.1 KW cell disrupter (Constant Systems) at 10 °C. The lysate was sequentially centrifuged at 600*g* for 10 min and 15,000*g* for 5 min. The supernatant was then centrifuged at 100,000*g* for 1 h at 4 °C using a Type 45 Ti rotor (Beckman-Coulter) to pellet cellular membranes. Pelleted membranes were homogenized in 20 mM HEPES pH 7.5, 500 mM NaCl, 15% (v/v) glycerol (MP Biochemicals) and 1% (w/v) LMNG (Anatrace), supplemented with a minimum of 2.5 U ml^−1^ benzonase and cOmplete protease inhibitor cocktail (EDTA-free). Membranes were solubilized in this buffer at 4 °C overnight, and the insoluble fraction removed by centrifugation at 142,000*g* for 4 h using a Type 70 Ti rotor (Beckman-Coulter). The clarified lysate was sequentially filtered using 5.0 and 0.45 μm PES membrane filters (Sartorius). Filtered lysate was applied to a Strep-Tactin XT Sepharose resin (Cytiva) pre-equilibrated into 20 mM HEPES pH 7.5, 500 mM NaCl and 15% (v/v) glycerol. The clarified lysate was bound to the resin in batch for 90 min at 4 °C with rotation, and the resin washed in 30 column volumes with wash buffer (20 mM HEPES pH 7.5, 500 mM NaCl, 0.05% (w/v) glyco-diosgenin (GDN) and 1 mM EDTA). The bound complex was eluted using wash buffer supplemented with 50 mM d-biotin (Merck, no. B4501). For analytical size-exclusion chromatography, 1% of pooled eluted material was size excluded using a Superose 6 Increase 10/300GL column (Cytiva) into 20 mM HEPES pH 7.5, 150 mM NaCl and 0.01% (w/v) GDN. Fractions collected were analysed by SDS–PAGE using Bolt 4–12% Bis-Tris Plus gels (Invitrogen, no. NW04125BOX), and protein bands were detected using a Pierce Silver Stain kit (Thermo Fisher, no. 24612). For the final complex preparation for cryo-EM analysis, affinity-purified protein was complexed with a tenfold molar excess of UCHT1 Fab fragment before a final size-exclusion purification in 10 mM HEPES pH 7.4, 150 mM NaCl and 0.02% (w/v) GDN.

### Cryo-EM sample preparation

Transmission electron microscopy UltrAuFoil R1.2/R1.3 Au 300 gold foil grids (QuantiFoil) were plasma cleaned immediately before sample vitrification. Purified γδ TCR–UCHT1 Fab complex was applied (3 μl) to the grids at a concentration of 5 mg ml^−1^ using a Vitrobot Mark IV (Thermo Fisher Scientific) at 4 °C, 100% humidity and blotted for 3 s at −2 blot force before vitrification in liquid ethane.

### Cryo-EM data collection parameters

Optimized grids were transferred for imaging to a Thermo Fisher Scientific Titan Krios transmission electron microscope operated at 300 kV, with a 50 µm C2 aperture, at a nominal magnification of ×105,000 in nanoprobe energy-filtering TEM mode corresponding to a pixel size of 0.82 Å. A Gatan K3 direct detection camera equipped with a Gatan Quantum energy filter was used alongside automatic data acquisition performed using ThermoScientific Smart EPU Software. Briefly, for the γδ TCR–UCHT1 Fab complex, 8.62 s exposures through a defocus range of −0.5 to −1.5 μm were dose fractioned into 60-frame videos collected in energy-filtered mode using a slit width of 10 eV and with a total dose of 60 e Å^–2^.

### Image processing and map generation

Following data collection as bias-only, LZW-compressed TIFFs, dose-fractionated videos were aligned, corrected for beam-induced motion, dose weighted and averaged within MotionCor2 (ref. ^41^). Estimation of CTF parameters was made using the CTFFIND 4.1.14 software package^42^. Automated particle picking was conducted using the real-time GPU accelerated-particle picking software Gautomatch v.0.53 (developed by K. Zhang: https://sbgrid.org/software/titles/gautomatch), in reference-free, cross-correlation picking mode. The resultant particles were extracted in RELION 4.0 (ref. ^43^) with a box size equivalent to 360 Å, downscaled to 60 pixels to expedite processing. The binned particle dataset was subjected to reference-free, two-dimensional classification using CryoSPARC v.4.2.0 software^44^, with iterative rounds of particle classification yielding high-resolution, two-dimensional classes. A curated particle subset was used for three-dimensional initial model generation using ab initio reconstruction with CryoSPARC. The resultant reconstruction was used for homogeneous refinement of a wider particle dataset using homogeneous refinement with CryoSPARC, with the refined particle coordinates subsequently re-extracted at 480 Å box size downscaled to 240 pixels in RELION 4.0. Heterogeneous refinement, further homogeneous refinement and ab initio classification were undertaken with CryoSPARC. The resultant particle subset was re-extracted at 480 Å unbinned box size using RELION 4.0, followed by Bayesian particle polishing following an additional non-uniform refinement with CryoSPARC. The polished particle set was next used for iterative non-uniform refinement with CryoSPARC to optimize per-particle defocus, group CTF refinement and three-dimensional autorefinement. A final three-dimensional classification and non-uniform refinement with CryoSPARC isolated a refined and sharpened consensus reconstruction at 3.01 Å. Throughout processing, global resolutions were calculated according to the gold-standard FSC criterion of 0.143. Local-resolution estimations were conducted with half-reconstructions as input maps in CryoSPARC. The 3.01 Å overall map for the γδ TCR–UCHT1 Fab complex was postprocessed using DeepEMhancer^45^ to enhance the protein signal, yielding noise-reduced and signal-enhanced maps.

To increase the signal in the TM region, we proceeded to repick particles using the machine learning-based picker TOPAZ v.0.2.5 (ref. ^46^) to increase the number of particles available for analysis. The above particle set resulting in the consensus map was used for training before picking the full dataset using the TOPAZ picker through the RELION 5.0 (ref. ^47^) wrapper. An approximate total of 9.9 million particles was extracted eight times binned in RELION 5.0, then further sorted using CryoSPARC two-dimensional classification and heterogeneous refinement, followed by homogeneous refinement, to retain around 1.5 million particles corresponding to the G83.C4 γδ TCR–UCHT1 Fab complex. These particles were then re-extracted and binned twice using the refined coordinates in RELION 5.0. The resulting particles were then imported back to CryoSPARC. Two rounds of ab inito classification were performed to retain 292,688 particles, which were then subjected to homogeneous and then non-uniform refinement to yield a Nyquist limited map at 3.35 Å. The resulting particles were then merged with the particle set that resulted in the consensus reconstruction. Duplicate particles were removed and the remaining particles then imported to CryoSPARC, wherein they were subjected to a round of homogeneous then heterogeneous refinement to retain 243,644 particles, followed by non-uniform refinement. These particles were further subjected to Bayesian polishing in RELION 5.0 and the polished particles were then extracted to a box size of 320 pixels. These particles were reimported to CryoSPARC for further refinement to yield a 3.30 Å map post CTF refinement, enabled during non-uniform refinement. A masked, three-dimensional classification focusing on the TM region and CD3 ECDs was performed on the CTF refined particle set. This resulted in 74,654 particles that exhibited reduced dynamics in the TM region. This subset of particles was subjected to a round of non-uniform refinement followed by local refinement to yield a 3.39 Å consensus map (using the gold-standard FSC 0.143 criterion) for the TM region.

### Atomic model building and refinement

Following finalization of the cryo-EM maps, crystal structures of the previously solved G83.C4 TCR (PDB 7LLI)^4^, αβ TCR (PDB 7PHR)^3^ and UCHT1 Fab (PDB 1XIW)^24^ were used as starting models for domain placement using rigid body refinement in Phenix (v.1.21.1)^48,49^. Using Coot (v.0.9.8.93)^50^, the domains and linkers were built iteratively before real-space refinement in Phenix, including calculation of model-to-map correlation statistics^48,49^. Regions and side-chains with poor density were removed and the final models validated using the Molprobity^51^ and PDB validation service server (https://validate-rcsb-1.wwpdb.org/). For the γδ TCR–UCHT1 Fab complex DeepEMhancer maps, *B*-factor-sharpened and -non-sharpened maps were utilized throughout model building and the DeepEMhancer post-processed maps were used for figure preparation. For the TM-focused analysis, *B*-factor-sharpened and -non-sharpened maps were utilized for model building and figure preparation. All structural figures were prepared using UCSF ChimeraX v.1.8 software^52^.

### Soluble MR1 production

Soluble MR1 and β_2_M were expressed and refolded with 5-OP-RU using previously established methods^53,54^. The 5-OP-RU ligand was generated in situ by the addition of 5-A-RU and methylglyoxal, as previously described^54^. Briefly, BL21 *Escherichia coli* inclusion bodies of MR1 and β_2_M, respectively, were refolded with ligand in 100 mM Tris-HCl pH 8.5, 2 mM EDTA, 5 M urea, 0.4 M arginine, 0.5 mM oxidized glutathione, 5 mM reduced glutathione and 1 mM phenylmethanesulfonyl fluoride. Refolded and ligand-loaded MR1 was then purified via size-exclusion chromatography and anion-exchange chromatography to yield homogeneous and pure protein.

### SLBs

Glass coverslips of 0.17 mm thickness were thoroughly cleaned with 1 M KOH and rinsed with Milli-Q water and placed in 100% ethanol before drying inside a fume hood. Following plasma cleaning, coverslips were adhered to eight-well silicon chambers (Ibidi, no. 80841). Supported lipid bilayers (SLBs) were prepared by vesicle extrusion of 1 mg ml^−1^ liposome solution^55^. The lipid composition of liposomes included 96.5% 1,2-dioleoyl-sn-glycero-3- phosphocholine (DOPC), 2% 1,2-dioleoyl-sn-glycero-3-[(*N*-(5-amino-1-carboxypentyl)iminodiacetic acid)succinyl] (nickel salt) (DGS-NTA(ni)), 1% B1,2-dioleoyl-sn-glycero-3-phosphoethanolamine-*N*-(cap biotinyl) (sodium salt) (Biotinyl-Cap-PE) and 0.5% 1,2-distearoyl-sn-glycero-3-phosphoethanolamine-*N*-[methoxy(poly-ethylene glycol)-5000] (ammonium salt) (PEG5000-PE) (mol%; all available from Avanti Polar Lipids (DOPC, no. 850375 C; DGS-NTA(Ni), no. 790404 C; Biotinyl-Cap-PE, no. 870273 C; PEG5000-PE, no. 880220 C). Extruded liposomes were added to eight-well chambers at a ratio of 1:5 with Milli-Q water (10 mM CaCl_2_) and incubated for 30 min at room temperature before gentle and repeated rinsing with PBS. By retaining about 200 µl of PBS in each well, disruption to SLBs was minimized during washing steps. Fluorescence recovery following photobleaching was used to examine the lateral mobility of freshly prepared SLBs by the addition of fluorescent streptavidin (Invitrogen, no. S11223)^55^. Excess Ca^2+^ ions on SLBs were removed with 0.5 mM EDTA, followed by gentle rinsing with Milli-Q water. The functionalized NTA groups in DGS-NTA(Ni) lipids were recharged by the addition of 1 mM NiCl_2_ solution to SLBs for 15 min. Excess Ni^2+^ ions were removed by repeated washing with PBS.

### T cell stimulation and immunostaining

The functionalized biotin groups on SLBs were coupled to 100 µg ml^−1^ streptavidin (Invitrogen, no. 434301), followed by a second coupling to biotinylated MR1(5-OP-RU) to yield a final concentration of 1–100 nM. NTA-functionalized lipids were coupled with 200 ng ml^−1^ His-tagged ICAM1 (Sino Biological, no. 10346-H08H). SLBs were repeatedly rinsed with PBS to remove excess unbound proteins. Before addition of the Jurkat TCR transductants, SLBs were incubated with warm RPMI culture medium (37 °C) for 30 min. T cells were stimulated on SLBs for 5 min at 37 °C, followed by immediate cell fixation with 4% paraformaldehyde (w/v) in PBS for 15 min at room temperature and then rinsed with PBS. Following fixation, T cells were permeabilized with 0.1% Triton X-100 (w/v) (Sigma-Aldrich) for 15 min and gently rinsed with PBS. Cells were blocked with 5% bovine serum albumin in PBS before immunostaining with anti-CD3ε-Alexa Fluor 647 (BioLegend, no. 300416, clone UCHT1, diluted 1:300) and anti-pCD3ζ-Alexa Fluor 568 (BD Biosciences, no. 558402, diluted 1:300) antibodies for 1 h at room temperature. Immunostaining was followed by multiple washes with PBS to remove excess unbound antibodies. A final fixation step was carried out using 4% paraformaldehyde (w/v) in PBS for 15 min. Finally, 0.1 µm TetraSpeck microspheres (Invitrogen, no. T7279) were embedded onto the lipid bilayers to allow for drift correction during super-resolution imaging.

### Single-molecule imaging with dSTORM

Imaging buffer, consisting of TN buffer (50 mM Tris-HCl pH 8.0 and 10 mM NaCl), the oxygen scavenger system GLOX (0.5 mg ml^−1^ glucose oxidase (Sigma-Aldrich, no. G2133), 40 mg ml^−1^ catalase (Sigma-Aldrich, no. C-100) and 10% w/v glucose) and 10 mM 2-aminoethanethiol (Sigma-Aldrich, no. M6500), was used for single-molecule imaging with direct stochastic optical reconstruction microscopy (dSTORM). Imaging sequences for dSTORM were acquired on a total internal reflection fluorescence microscope (Nikon N-STORM 5.0) equipped with a ×100/1.49 numerical aperture oil-immersion objective and lasers (405, 473, 561 and 640 nm). Time series of 10,000 frames were acquired per sample, per channel (640 or 561 nm laser channel with continuous low-power 405 nm illumination), with an exposure time of 30 ms in total internal reflection fluorescence mode. For dual-colour acquisition, the higher-wavelength channel (640 nm laser for Alexa Fluor 647) was acquired first, followed by the channel with shorter wavelength (561 nm laser for Alexa Fluor 568) using a sCMOS camera (Hamamatsu Orca-Flash 4.0 V3). Image processing, including fiducial marker-based drift correction, two-channel alignment and generation of *x*–*y* particle coordinates for each localization was carried out using NIS-Elements AR software (v.5.2).

### Cluster analysis of single-molecule images

For quantification of cluster parameters in single-molecule images, we used an algorithm^56^ that utilizes density-based spatial clustering with noise analysis implemented in MATLAB. Here, we predetermine the minimum number of neighbours (minimum points, 3) and the radius that they occupy (*r* = 20 nm). A combined cluster detection and colocalization analysis was performed that quantifies both spatial distribution and the degree of colocalization (DoC) of two proteins (Clus–DoC)^56^. This analysis relies on generating density gradients for each individual localization by calculating the number of molecules captured from both channels with increasing circle radius (*r* = 20 nm). These density gradients are then normalized to the density at the maximum radius for channels 1 and 2, respectively. The resulting two types of distribution generated for each channel are then compared by calculating the rank correlation coefficient using Spearman correlation, in which the local coefficient is measured by a value proportional to the distance of the nearest neighbour. Accordingly, each localization was assigned with a DoC score ranging from +1 (indicating colocalization) to −1 (indicating segregation), with 0 indicating random distribution. As previously discussed^55^, the threshold for DoC is a user-defined variable that can be optimized for different experimental conditions. Here, the DoC threshold was set to 0.1, above which the values were taken to represent the fraction of cluster colocalization events captured between the two fluorescence channels for the TCR (Alexa Fluor 647) and pCD3ζ (Alexa Fluor 568), with increases in cluster colocalization reflecting increased TCR signalling.

### FLIM–FRET assay

For the detection of FLIM–FRET between TCR and MR1 monomers in solution, Jurkat T cells expressing the TCR of interest were stained with Alexa 647-conjugated anti-CD3 UCHT1 Fab and Atto 594 maleimide-conjugated MR1-5-OP-RU, utilized as fluorescence acceptor and donor, respectively. Approximately 10^5^ cells were stained with 100 ng of anti-CD3 Alexa 647-labelled Fabs in a staining volume of 30 µl for 20 min at 4 °C in ice-cold, phenol-free RPMI (imaging buffer). Following the incubation period, two washing steps were performed using imaging buffer to remove excess Fab. Cells were subsequently stored at 4 °C in imaging buffer before imaging. FLIM–FRET experiments were conducted on a Leica Stellaris 5 Confocal Microscope equipped with a white-light laser, HyD-S detectors and an HC PL APO ×63/1.40 numerical aperture oil-immersion objective lens. FRET events were captured using TauInteraction mode, with fluorescence images acquired in frame sequential mode. FRET imaging was undertaken using 10% of the 602 nm laser line (for Atto 594) and 10% of the 653 nm laser line (for Alexa 647) with the HyD-S2 detector (612–650 nm) and HyD-S3 detector (663–829 nm), respectively. TauContrast and TauInteraction modes were enabled for both detectors, with the operating mode set to counting. The fluorescent donor and acceptor fluorescence lifetimes were calculated using donor-alone and acceptor-alone controls, which closely match previously reported fluorescence lifetime values for the Atto 594 donor (3.5 ns) and Alexa 647 acceptor (1.0 ns). These values were then manually selected under TauInteraction mode. For imaging, anti-CD3 UCHT Fab-AF647-stained Jurkat T cells were immobilized on a cleaned glass coverslip (0.17 mm thickness) coated with 0.1% (w/v) poly l-lysine (Merck, no. P8920) and adhered to eight-well silicon chambers (Ibidi, no. 80841). Following a 10 min incubation with warm imaging buffer to reach 37 °C, imaging was initiated following the addition of 1 ng µl^−1^ MR1-5-OP-RU-Atto 594 monomers to each chamber containing immunostained T cells. Imaging was completed within 10 min of the addition of the donor. FLIM–FRET heatmaps were generated using LAS-X v.5.2.2 imaging software (Leica) and the Fiji image processing package within ImageJ v.1.54 f software, with FLIM–FRET efficiency reported as a percentage using the mean weighted TauInteraction output value generated per region of interest using LAS-X v.5.2.2 imaging software.

### Cluster analysis code availability

The link to the cluster analysis algorithm used in this study is available at the GitHub repository link (https://github.com/PRNicovich/ClusDoC)^56^.

### Jurkat T cell activation

For cell-based T cell activation assays, 1 × 10^5^ Jurkat TCR transductants were cocultured for either 20 or 4 h with 5 × 10^4^ C1R.WT cells treated with serial dilutions of 5-OP-RU (1.0–0.0001 nM). Following the coculture incubation period, cells were harvested, washed with PBS and stained with anti-CD20-PE.Cy7 (BioLegend, no. 302312, 1:300 dilution), anti-CD69-Pacific Blue (BioLegend, no. 310920, 1:300 dilution) and anti-CD3ε-FITC (BioLegend, no. 300440, 1:300 dilution). For plate-bound OKT3-based activation assays, 96-well flat-bottomed plates were first coated with 10 µg µl^−1^ OKT3 (TONBO Biosciences, no. 70-0037-U100, 1:100 dilution) overnight at 4 °C. Plates were washed three times with PBS before the addition of 2.5 × 10^5^ cells per well. CD69 expression was analysed using flow cytometry 4 h post stimulation using anti-CD69-Pacific Blue. For activation assays in the presence of UCHT1 Fab, cells were first incubated with a range of concentrations of UCHT1 Fab (0–50 µg µl^−1^) for 30 min. UCHT1 Fab-labelled cells were then cocultured with ligand-treated C1R cells for 4 h and cells stained as before. Flow cytometry data were acquired using a Becton Dickinson LSRFortessa Cell Analyser. To examine CD69 expression in Jurkat T cells, fluorescence activated cell-sorting plots were gated on the CD20-negative population following elimination of dead cells and doublets. All flow cytometry data were analysed using the CytoExploreR package in R (v.12.1).

### Mass spectrometry

A 50 µl solution of 5 µg µl^−1^ purified protein (about 250 µg) in HBS containing 0.05% GDN was made up to a final volume of 100 µl by the addition of 50 µl of water, and was extracted together with a positive and a negative blank control. These controls each comprised 100 µl of water with the addition to the positive control of 387 ng of cholesterol (1.068 µl of 362 ng µl^−1^ in 2:1 chloroform:methanol v/v). Samples were then prepared using a modified Folch extraction and analysed by liquid chromatography–mass spectrometry as described previously^57^.

### Constructs for WT and chimeric TCR expression

Sequences for the expression of all constructs encoding WT and chimeric AF-7 and G83.C4 TCRs are included in Supplementary Table 1.

### CP length determination

Sequences for all functional mammalian genes were obtained from the IMGT database^58^. Where multiple alleles exist, those first listed (that is, the allele ending in *01) were selected. Sequences were aligned using Muscle^59^, and sequences matching the human *TRGC-* or *TRDC*-encoded CPs were extracted and used to calculate CP length.

### Statistical analyses

When comparing multiple groups, statistical analysis and *P* values were calculated using one-way analysis of variance in GraphPad Prism software (v.9.5.1). Error bars represent s.e.m. or s.d. as specified.

### Ethics and inclusion statement

The authors confirm that the research included local researchers throughout the research process—that is, during the design of the study, its implementation and with respect to authorship. We also confirm that the roles and responsibilities were agreed amongst the collaborators ahead of the research and that capacity-building plans for each group of local researchers were discussed. The study did not require local ethics review because it did not involve animal experiments or the use of human tissue.

### Reporting summary

Further information on research design is available in the Nature Portfolio Reporting Summary linked to this article.

## Online content

Any methods, additional references, Nature Portfolio reporting summaries, source data, extended data, supplementary information, acknowledgements, peer review information; details of author contributions and competing interests; and statements of data and code availability are available at 10.1038/s41586-024-07920-0.

## Supplementary information

Supplementary Information Supplementary Fig. 1 and Table 1.

Reporting Summary

## Figures and Tables

**Extended Data Table 1:**
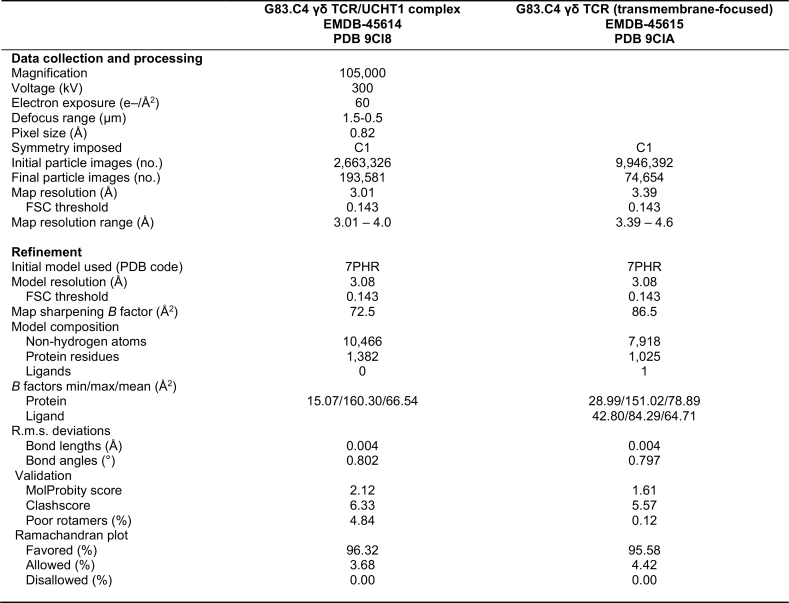
Cryo-EM data collection, refinement and validation statistics

## Data Availability

The atomic coordinates for the G83.C4 γδ TCR–UCHT1 Fab and G83.C4 γδ TCR–CD3 TM-focused complexes have been deposited at the Protein Data Bank under accession codes 9CI8 and 9CIA, respectively (https://www.ebi.ac.uk/pdbe/). All *B*-factor-sharpened, -non-sharpened, half-maps and postprocessed DeepEMhancer cryo-EM maps for the G83.C4 γδ TCR–UCHT1 Fab and G83.C4 γδ TCR–CD3 TM-focused complexes have been deposited at the Electron Microscopy Data Bank under accession codes EMD-45614 and EMDB-45615, respectively (https://www.ebi.ac.uk/emdb/). The previously published model of an αβ TCR used for initial model building is available at the Protein Data Bank under accession no. PDB 7PHR. Expression constructs used in this study will be made available via a public repository (https://www.addgene.org/).

## Code availability

Neither custom code nor mathematical algorithms were used for this study.
