# Supplementary material for: Structure of a fully assembled γδ T cell antigen receptor
Source: Nature. 2024 Aug 15;634(8034):729–36. doi: 10.1038/s41586-024-07920-0 (PMC11485255; doi:10.1038/s41586-024-07920-0)
Supplement: Supplementary file 1 — Supplementary Fig. 1 and Table 1. [file 41586_2024_7920_MOESM1_ESM.pdf]

nature portfolio

https://doi.org/10.1038/s41586-024-07920-0

Supplementary information

Structure of a fully assembled γδ T cell antigen receptor

In the format provided by the authors and unedited

Supplementary information

Structure of a fully assembled γδ T-cell antigen receptor

Benjamin S. Gully, João Ferreira Fernandes, Sachith D. Gunasinghe, Mai T. Vuong,
Yuan Lui, Michael T. Rice, Liam Rashleigh, Chan-sien Lay, Dene R. Littler, Sumana Sharma,
Ana Mafalda Santos, Hariprasad Venugopal, Jamie Rossjohn & Simon J. Davis

Supplementary Figure 1

Supplementary Table 1

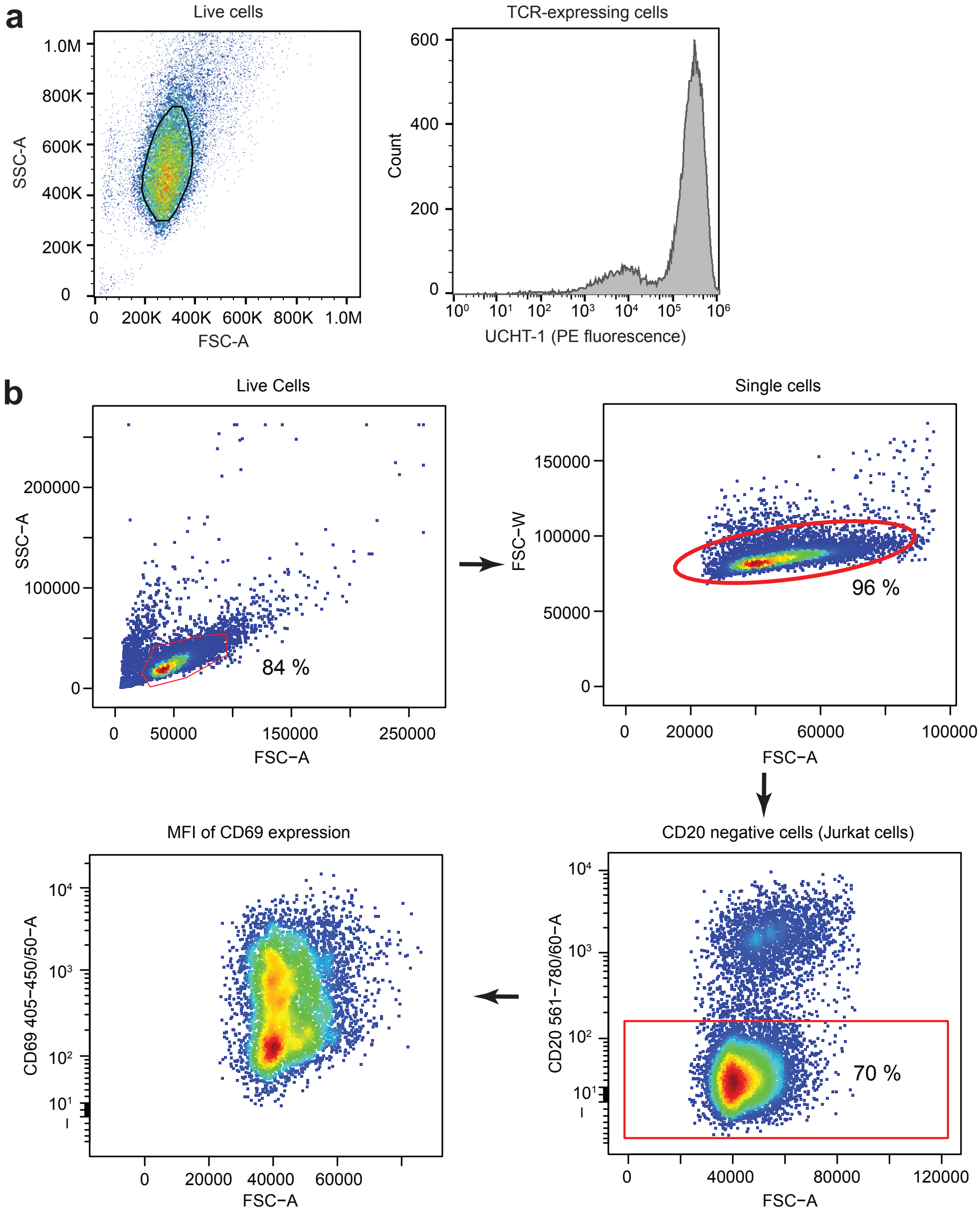

Supplementary Figure 1 | Gating strategies for flow cytometry. a, Representative gating strategy for surface molecule staining of the Jurkat T-cell lines. **b**, Representative gating strategy for measurements of CD69 expression in the T-cell activation assay.

Supplementary Table 1 | Sequences of the protein constructs used in this study

Name	Protein sequence
1. **G83.C4 SpyT-TCRγCD3δε_GFP2**<br><br>(For expression and purification of the Twin-StrepTag tagged complex, together with 2.)	MLLALALLLAFLPPASQKMGRGVPHIVMVDAYKRYK<br>SGGGSGENLYFQGGSGESGSGSSNLEGRTKSVTR<br>PTGSSAVITCDLPVENAVYTHWYLHQEGKAPQRLLY<br>YDSYNSRVVLESGISREKYHTYASTGKSLKFILENLIE<br>RDSGVYYCATWDYKKLFGSGTTLVVTDKQLDADVS<br>PKPTIFLPSIAETKLQKAGTYLCLLEKFFPDVIKIHWQE<br>KKSNTILGSQEGNTMKTNDTYMKFSWLTVPEKSLDK<br>EHRCIVRHENNKNGVDQEIIFPPIKTDVITMDPKDNCS<br>KDANDTLLLQLTNTSAYYMYLLLLLKSVVYFAIITCCLL<br>RRTAFCCNGEKSGSGQCTNYALLKLAGDVESNPGP<br>MEHSTFLSGLVLATLLSQVSPFKIPIEELEDRVFVNCN<br>TSITWVEGTVGTLLSDITRLDLGKRILDPRGIYRCNGT<br>DIYKDKESTVQVHYRMCQSCVELDPATVAGIIVTDVI<br>ATLLLALGVFCFAGHETGRLSGAADTQALLRNDQVY<br>QPLRDRDDAQYSHLGGNWARNKGSGEGRGSLLTC<br>GDVEENPGPMQSGTHWRVLGLCLLSVGVWGQDGN<br>EEMGGITQTPYKVSISGTTVILTCPQYPGSEILWQHN<br>DKNIGGDEDDKNIGSDEDHLSLKEFSELEQSGYYVC<br>YPRGSKPEDANFYLYLRARVCENCMEMDVMSVATIV<br>IVDICITGGLLLLVYYWSKNRKAKAKPVTRGAGAGGR<br>QRGQNKERPPPVPNPDYEPIRKGQRDLYSGLNQRR<br>IGDPPVATMVSKGEELFTGVVPILVELDGDVNGHKFS<br>VSGEGEGDATYGKLTLKFICTTGKLPVPWPTLVTTLS<br>YGVQCFSRYPDHMKQHDFFKSAMPEGYVQERTIFF<br>KDDGNYKTRAEVKFEGDTLVNRIELKGIDFKEDGNIL<br>GHKLEYNYNSHNVYIMADKQKNGIKVNFKIRHNIEDG<br>SVQLADHYQQNTPIGDGPVLLPDNHYLSTQSALSKD<br>PNEKRDHMVLLEFVTAAGITLGMDELYK
2. **G83.C4 TCRδCD3ζγ_Strep2**<br><br>(For expression and purification of the Twin-StrepTag tagged complex, together with 1.)	MILTVGFSFLFFYRGTLCDKVTQSSPDQTVASGSEV<br>VLLCTYDTVYSNPDLFWYRIRPDYSFQFVFYGDNSR<br>SEGADFTQGRFSVKHILTQKAFHLVISPVRTEDSATY<br>YCATRLWLGDPHTDKLIFGKGTRVTVEPRSQPHTKP<br>SVFVMKNGTNVACLVKEFYPKDIRINLVSSKKITEFDP<br>AIVISPSGKYNAVKLGKYEDSNSVTCSVQHDNKTVH<br>STDFEVKTDSTDHVKPKETENTKQPSKSCHKPKAIV<br>HTEKVNMMSLTVLGLRMLFAKTVAVNFLLTAKLFFLG<br>SGQCTNYALLKLAGDVESNPGPMKWKALFTAAILQA<br>QLPITEAQSFGLLDPKLCYLLDGILFIYGVILTALFLRV<br>KFSRSADAPAYQQGQNQLYNELNLGRREEYDVLDK<br>RRGRDPEMGGKPQRRKNPQEGLYNELQKDKMAEA<br>YSEIGMKGERRRGKGHDGLYQGLSTATKDTYDALH<br>MQALPPRGSGEGRGSLLTCGDVEENPGPMEQGKG<br>LAVLILAIILLQGTLAQSIKGNHLVKVYDYQEDGSVLLT<br>CDAEAKNITWFKDGKMIGFLTEDKKKWNLGSNAKDP<br>RGMYQCKGSQNKSKPLQVYYRMCQNCIELNAATIS<br>GFLFAEIVSIFVLAVGVYFIAGQDGVRQSRASDKQTL<br>LPNDQLYQPLKDREDDQYSHLQGNQLRRNGSGSA<br>WSHPQFEKGGGSGGGSGGSAWSHPQFEK
3. **AF-7 TCR-α chain**<br><br>(For expression of the AF-7 TCR in Jurkat T-cells, together with 4.)	MWGVFLLYVSMKMGGTTGQNIDQPTEMTATEGAIV<br>QINCTYQTSGFNGLFWYQQHAGEAPTFLSYNVLDGL<br>EEKGRFSSFLSRSKGYSYLLLKELQMKDSASYLCAV<br>KDSNYQLIWGAGTKLIIKPDIQNPDPAVYQLRDSKSS<br>DKSVCLFTDFDSQTNVSQSKDSDVYITDKTVLDMRS<br>MDFKSNSAVAWSNKSDFACANAFNNSIIPEDTFFPS<br>PESSCDVKLVEKSFETDTNLNFQNLSVIGFRILLLKVA<br>GFNLLMTLRLWSS
4. **AF-7 TCR-β chain**<br><br>(For expression of the AF-7 TCR in Jurkat T-cells, together with 3.)	MGILPSPGMPALLSLVSLLSVLLMGCVAETSYPYDVP<br>DYAGGSGTSNAGVTQTPKFQVLKTGQSMTLQCAQD<br>MNHNSMYWYRQDPGMGLRLIYYSASEGTTDKGEVP<br>NGYNVSRLNKREFSLRLESAAPSQTSVYFCASSVWT<br>GEGSGELFFGEGSRLTVLEDLKNVFPPKVAVFEPSE

	AEISHTQKATLVCLATGFYPDHVELSWWVNGKEVHS<br>GVSTDPQPLKEQPALNDSRYCLSSRLRVSATFWQN<br>PRNHFRCQVQFYGLSENDEWTQDRAKPVTQIVSAE<br>AWGRADCGFTSESYQQGVLSATILYEILLGKATLYAV<br>LVSALVLMAMVKRKDSRG
5. **G83.C4 TCR-γ chain**<br><br>(For expression of the G83.C4 TCR in Jurkat T-cells, together with 6.)	MLLALALLLAFLPPASQKTSYPYDVPDYAGGSGTSS<br>SNLEGRTKSVTRPTGSSAVITCDLPVENAVYTHWYL<br>HQEGKAPQRLLYYDSYNSRVVLESGISREKYHTYAS<br>TGKSLKFILENLIERDSGVYYCATWDYKKLFGSGTTL<br>VVTDKQLDADVSPKPTIFLPSIAETKLQKAGTYLCLLE<br>KFFPDVIKIHWQEKKSNTILGSQEGNTMKTNDTYMKF<br>SWLTVPEKSLDKEHRCIVRHENNKNGVDQEIIFPPIK<br>TDVITMDPKDNCSKDANDTLLLQLTNTSAYYMYLLLL<br>LKSVVYFAIITCCLLRRTAFCCNGEKS
6. **G83.C4 TCR-δ chain**<br><br>(For expression of the G83.C4 TCR in Jurkat T-cells, together with 5.)	MILTVGFSFLFFYRGTLCDKVTQSSPDQTVASGSEV<br>VLLCTYDTVYSNPDLFWYRIRPDYSFQFVFYGDNSR<br>SEGADFTQGRFSVKHILTQKAFHLVISPVRTEDSATY<br>YCATRLWLGDPHTDKLIFGKGTRVTVEPRSQPHTKP<br>SVFVMKNGTNVACLVKEFYPKDIRINLVSSKKITEFDP<br>AIVISPSGKYNAVKLGKYEDSNSVTCSVQHDNKTVH<br>STDFEVKTDSTDHVKPKETENTKQPSKSCHKPKAIV<br>HTEKVNMMSLTVLGLRMLFAKTVAVNFLLTAKLFFL
7. **AF-7 V-α/C-δ chain**<br><br>(For expression of the chimeric AF-7 TCR in Jurkat T-cells, together with 8; C-δ chain sequence underlined.)	MWGVFLLYVSMKMGGTTGQNIDQPTEMTATEGAIV<br>QINCTYQTSGFNGLFWYQQHAGEAPTFLSYNVLDGL<br>EEKGRFSSFLSRSKGYSYLLLKELQMKDSASYLCAV<br>KDSNYQLIWGAGTKLIIKPDSQPHTKPSVFVMKNGTN<br>VACLVKEFYPKDIRINLVSSKKITEFDPAIVISPSGKYN<br>AVKLGKYEDSNSVTCSVQHDNKTVHSTDFEVKTDST<br>DHVKPKETENTKQPSKSCHKPKAIVHTEKVNMMSLT<br>VLGLRMLFAKTVAVNFLLTAKLFFL
8. **AF-7 V-β/C-γ chain**<br><br>(For expression of the chimeric AF-7 TCR in Jurkat T-cells, together with 7; C-γ chain sequence underlined.)	MGILPSPGMPALLSLVSLLSVLLMGCVAETSYPYDVP<br>DYAGGSGTSNAGVTQTPKFQVLKTGQSMTLQCAQD<br>MNHNSMYWYRQDPGMGLRLIYYSASEGTTDKGEVP<br>NGYNVSRLNKREFSLRLESAAPSQTSVYFCASSVWT<br>GEGSGELFFGEGSRLTVLEDKQLDADVSPKPTIFLPS<br>IAETKLQKAGTYLCLLEKFFPDVIKIHWQEKKSNTILG<br>SQEGNTMKTNDTYMKFSWLTVPEKSLDKEHRCIVR<br>HENNKNGVDQEIIFPPIKTDVITMDPKDNCSKDANDT<br>LLLQLTNTSAYYMYLLLLLKSVVYFAIITCCLLRRTAFC<br>CNGEKS
9. **G83.C4 V-γ/C-β chain**<br><br>(For expression of the chimeric G83.C4 TCR in Jurkat T-cells, together with 10; C-β chain sequence underlined.)	MLLALALLLAFLPPASQKTSYPYDVPDYAGGSGTSS<br>SNLEGRTKSVTRPTGSSAVITCDLPVENAVYTHWYL<br>HQEGKAPQRLLYYDSYNSRVVLESGISREKYHTYAS<br>TGKSLKFILENLIERDSGVYYCATWDYKKLFGSGTTL<br>VVTDLKNVFPPKVAVFEPSEAEISHTQKATLVCLATG<br>FYPDHVELSWWVNGKEVHSGVSTDPQPLKEQPALN<br>DSRYCLSSRLRVSATFWQNPRNHFRCQVQFYGLSE<br>NDEWTQDRAKPVTQIVSAEAWGRADCGFTSESYQQ<br>GVLSATILYEILLGKATLYAVLVSALVLMAMVKRKDSR<br>G
10. **G83.C4 V-δ/C-α chain**<br><br>(For expression of the chimeric G83.C4 TCR in Jurkat T-cells, together with 9; C-α chain sequence underlined.)	MILTVGFSFLFFYRGTLCDKVTQSSPDQTVASGSEV<br>VLLCTYDTVYSNPDLFWYRIRPDYSFQFVFYGDNSR<br>SEGADFTQGRFSVKHILTQKAFHLVISPVRTEDSATY<br>YCATRLWLGDPHTDKLIFGKGTRVTVEPRIQNPDPA<br>VYQLRDSKSSDKSVCLFTDFDSQTNVSQSKDSDVYI<br>TDKTVLDMRSMDFKSNSAVAWSNKSDFACANAFNN<br>SIIPEDTFFPSPESSCDVKLVEKSFETDTNLNFQNLSV<br>IGFRILLLKVAGFNLLMTLRLWSS
