# Supplementary material for: Structure of a fully assembled γδ T cell antigen receptor
Source: Nature. 2024 Aug 15;634(8034):729–36. doi: 10.1038/s41586-024-07920-0 (PMC11485255; doi:10.1038/s41586-024-07920-0)
Supplement: Supplementary file 2 — Reporting Summary [file 41586_2024_7920_MOESM2_ESM.pdf]

nature portfolio

Corresponding author(s): Davis SJ, Rossjohn J

Last updated by author(s): Jul 26, 2024

Reporting Summary

Nature Portfolio wishes to improve the reproducibility of the work that we publish. This form provides structure for consistency and transparency in reporting. For further information on Nature Portfolio policies, see our Editorial Policies and the Editorial Policy Checklist.

Statistics

For all statistical analyses, confirm that the following items are present in the figure legend, table legend, main text, or Methods section.

n/a	Confirmed	
☐	☒	The exact sample size (*n*) for each experimental group/condition, given as a discrete number and unit of measurement
☐	☒	A statement on whether measurements were taken from distinct samples or whether the same sample was measured repeatedly
☐	☒	The statistical test(s) used AND whether they are one- or two-sided<br>*Only common tests should be described solely by name; describe more complex techniques in the Methods section.*
☒	☐	A description of all covariates tested
☒	☐	A description of any assumptions or corrections, such as tests of normality and adjustment for multiple comparisons
☐	☒	A full description of the statistical parameters including central tendency (e.g. means) or other basic estimates (e.g. regression coefficient) AND variation (e.g. standard deviation) or associated estimates of uncertainty (e.g. confidence intervals)
☐	☒	For null hypothesis testing, the test statistic (e.g. *F*, *t*, *r*) with confidence intervals, effect sizes, degrees of freedom and *P* value noted<br>*Give P values as exact values whenever suitable.*
☒	☐	For Bayesian analysis, information on the choice of priors and Markov chain Monte Carlo settings
☒	☐	For hierarchical and complex designs, identification of the appropriate level for tests and full reporting of outcomes
☒	☐	Estimates of effect sizes (e.g. Cohen's *d*, Pearson's *r*), indicating how they were calculated

Our web collection on statistics for biologists contains articles on many of the points above.

Software and code

Policy information about availability of computer code

Data collection	Smart EPU software (ThermoFisher).
Data analysis	R (v12.10); GraphPad Prism (v9.5.1); Gautomatch (v0.53); Coot (v0.9.8.93); RELION (v4.0 and v5.0); MotionCor (version MotionCor2); CryoSPARC (v4.2.0); DeepEMhancer; UCSF ChimeraX (v1.8); TOPAZ (v0.2.5); NIS-Elements AR software (v5.2); LAS-X software (v5.2.2); ImageJ (v1.54f); CTTFIND (v4.1.14); Clus-DoC; FlowJo (v10.8.1).

For manuscripts utilizing custom algorithms or software that are central to the research but not yet described in published literature, software must be made available to editors and reviewers. We strongly encourage code deposition in a community repository (e.g. GitHub). See the Nature Portfolio guidelines for submitting code & software for further information.

Data

Policy information about availability of data

All manuscripts must include a data availability statement. This statement should provide the following information, where applicable:

- Accession codes, unique identifiers, or web links for publicly available datasets
- A description of any restrictions on data availability
- For clinical datasets or third party data, please ensure that the statement adheres to our policy

The atomic coordinates for the G83.C4 γδ TCR/UCHT1 Fab and G83.C4 γδ TCR/CD3 TM-focused complexes have been deposited at the Protein Databank under accession codes 9CI8 and 9CIA, respectively (https://www.ebi.ac.uk/pdbe/).

All the B-factor sharpened, non-sharpened, half-maps and post-processed DeepEMhancer cryo-EM maps for the G83.C4 γδ TCR/UCHT1 Fab and G83.C4 γδ TCR/CD3 TM-focused complexes have been deposited at the Electron Microscopy Data Bank under accession codes EMD-45614 and EMDB-45615, respectively (https://www.ebi.ac.uk/emdb/).

The previously published model of the αβTCR used for initial model building is available on the Protein Data Bank under accession number PDB 7PHR.

Research involving human participants, their data, or biological material

Policy information about studies with human participants or human data. See also policy information about sex, gender (identity/presentation), and sexual orientation and race, ethnicity and racism.

Reporting on sex and gender	N/A
Reporting on race, ethnicity, or other socially relevant groupings	N/A
Population characteristics	N/A
Recruitment	N/A
Ethics oversight	N/A

Note that full information on the approval of the study protocol must also be provided in the manuscript.

Field-specific reporting

Please select the one below that is the best fit for your research. If you are not sure, read the appropriate sections before making your selection.

☒ Life sciences ☐ Behavioural & social sciences ☐ Ecological, evolutionary & environmental sciences

For a reference copy of the document with all sections, see nature.com/documents/nr-reporting-summary-flat.pdf

Life sciences study design

All studies must disclose on these points even when the disclosure is negative.

Sample size	Sample size for cryo-EM was determined as required to generate a suitable resolution. For cell-based assays, no calculations were performed to determine sample size. Cells used in these assays were derived from homogenous cell lines, and the number of cells used was chosen to ensure enough cells could be acquired by flow cytometry or light microscopy to adequately sample expression levels or fluorescence signals.
Data exclusions	No data were excluded from the analysis.
Replication	All experiments except the tetramer-staining of the MR1-expressing cell line was done at least in triplicates. The number of biological and technical replicates are described in the figure legends.
Randomization	All cryo-EM data was acquired from a single homogenous purified protein and cell-based assays were not conducted on groups with variable individuals, so randomization was not required.
Blinding	All cryo-EM data was acquired from a single homogenous purified protein and cell-based assays were not conducted on groups with variable individuals, so blinding was not applicable. Negative and positive controls were included in experiments and all measurements were carried out at the same time for a given experiment.

Reporting for specific materials, systems and methods

We require information from authors about some types of materials, experimental systems and methods used in many studies. Here, indicate whether each material, system or method listed is relevant to your study. If you are not sure if a list item applies to your research, read the appropriate section before selecting a response.

Materials & experimental systems

n/a	Involved in the study	
☐	☒	Antibodies
☐	☒	Eukaryotic cell lines
☒	☐	Palaeontology and archaeology
☒	☐	Animals and other organisms
☒	☐	Clinical data
☒	☐	Dual use research of concern
☒	☐	Plants

Methods

n/a	Involved in the study	
☒	☐	ChIP-seq
☐	☒	Flow cytometry
☒	☐	MRI-based neuroimaging

Antibodies

Antibodies used	Commercial antibodies were used for testing the transduced cell lines.<br><br>Anti-human γδTCR PE (clone B1, Biolegend 331210, diluted 1:40);<br>Anti-human CD3ε PE (clone UCHT1, Biolegend 300408, 1:10)<br>Anti-human CD3ε FITC (clone UCHT1, Biolegend 300400, 1:300);<br>Anti-human CD3ε AF647 (clone UCHT1, Biolegend 300416, 1:300);<br>Anti-human pCD3ζ AF568 (BD Biosciences 558402, 1:300);<br>Anti-human CD20 PE/Cy7 (clone 2H7, Biolegend 302312, 1:300);<br>Anti-human CD69 Pacific Blue (clone FN50, Biolegend 310920, 1:300);<br>Purified anti-human CD3ε (OKT3, TONBO Biosciences, 70-0037-U100, 1:100).
Validation	Antibodies purchased from Biolegend were validated by the supplier, using flow cytometry analysis of immunofluorescent staining. The purified OKT3 antibody (TONBO Biosciences) was not validated by the supplier.

Eukaryotic cell lines

Policy information about cell lines and Sex and Gender in Research

Cell line source(s)	Jurkat (clone E6-1), C1R and HEK293T cells were obtained from ATCC. CHO-K1 cells (D28-W1) were obtained from Lonza Biologicals. FreeStyle CHO-S cells were obtained from ThermoFisher.
Authentication	Cell lines obtained from ATCC were authenticated by the supplier by STR profiling. CHO-S cells were obtained from ThermoFisher, but the supplier does not provide information on the authentication process. The CHO-K1 cell line was obtained from Lonza Biologicals in 1988 and we no longer hold records of its authentication.
Mycoplasma contamination	Cells were found to be negative for mycoplasma contamination using both enzymatic assays and microscopy-based assays, conducted on a monthly basis.
Commonly misidentified lines (See ICLAC register)	Commonly misidentified lines were not used in this study.

Plants

Seed stocks	N/A
Novel plant genotypes	N/A
Authentication	N/A

Flow Cytometry

Plots

Confirm that:

☒ The axis labels state the marker and fluorochrome used (e.g. CD4-FITC).

☒ The axis scales are clearly visible. Include numbers along axes only for bottom left plot of group (a 'group' is an analysis of identical markers).

☒ All plots are contour plots with outliers or pseudocolor plots.

☒ A numerical value for number of cells or percentage (with statistics) is provided.

Methodology

Sample preparation	For all experiments, cells were harvested from culture and washed twice in PBS. Cells were stained with stated antibodies (see Methods) for 1h at 4oC. Cells were washed in PBS twice.
Instrument	For activation assays, data was analysed using BD Fortessa II, BD XDP machines. For CHO-S cell labelling, cells were analysed using an Attune NxT system.
Software	For activation assays, analysis was performed using the CytoExploreR package in R. For CHO-S cell labelling, analysis was performed using FlowJo (v10.8.1).
Cell population abundance	At least 10,000 cells were collected for all samples.
Gating strategy	For T-cell activation assays in co-culture: FSC-A/SSC-A > FSC-A/FSC-W > CD20 (-) population > CD69 expression.<br><br>For testing surface protein expression: FSC-A/SSC-A > fluorescence expression.

☒ Tick this box to confirm that a figure exemplifying the gating strategy is provided in the Supplementary Information.
